# Techno-structural and 3-D geometric morphometric analysis applied for investigating the variability of Holocene unifacial tools in tropical Central Brazil

**DOI:** 10.1371/journal.pone.0315746

**Published:** 2025-01-02

**Authors:** Marina González-Varas, Antoine Lourdeau, Letícia Gonçalves, Rafael Lemos de Souza, Diego Teixeira Mendes, Tatyana Beltrão de Oliveira, Gustavo Furlaneto Silva, Hubert Forestier, Rolando Romero, Antonio Pérez-Balarezo

**Affiliations:** 1 Département Homme & Environnement, Muséum National d’Histoire Naturelle, UMR 7194 HNHP, Équipe PRÉTROP, Musée de l’Homme, Paris, France; 2 Institut Français d’Etudes Andines (IFEA), Lima, Perú; 3 Instituto Goiano de Pré-História e Antropologia (IGPA), Pontificia Universidade Católica de Goiás (PUC-GO), Goiânia, Brasil; 4 Museu Antropológico da Universidade Federal da Goiás (UFG), Goiânia, Brasil; 5 Museu de Arqueologia e Etnologia (MAE), Universidade de São Paulo (USP), São Paulo, Brasil; 6 Faculdade de Ciências Sociais, Universidade Federal da Goiás (UFG), Goiânia, Brasil; 7 Universidad Tecnológica del Perú, Chiclayo, Lima, Perú; 8 Departamento Académico de Humanidades, Pontificia Universidad Católica del Perú (PUCP), Lima, Perú; 9 Grupo de Investigación en Poblamiento Inicial de las Américas (GIPAM) de la Pontificia Universidad Católica del Perú (PUCP), Lima, Perú; Universidad Nacional de la Plata Facultad de Ciencias Naturales y Museo, ARGENTINA

## Abstract

During the transition from the Pleistocene to the Holocene and in the early Holocene period, hunter-gatherer communities across tropical South America deployed a range of technological strategies to adapt to diverse environmental conditions. This period witnessed a rich tapestry of technological practices, from enduring, widely disseminated tools to local and sporadically utilized technologies, shaping a multifaceted landscape of technological traditions. Lithic technology during this period was mainly marked by localized sourcing of raw materials, the use of multifunctional tools, a variety of projectile point designs, and the frequently utilization of unifacial shaping technology. In tropical Central Brazil, the Itaparica technocomplex, with unique unifacial lithic tools like limaces, is a pivotal innovation from the Late Pleistocene through the Holocene. However, the factors influencing their morphological and structural variability remain largely unexplored, obscuring our understanding of their ergonomics and their role as mediators between humans and tropical environments. This study hypothesizes that the variability observed within and among unifacial tools from the GO-Ni sites in Central Brazil is a result of a combination of factors, including raw material availability and functional and ergonomic requirements. To test this hypothesis, a study of 67 unifacial tools from this region was conducted, employing techno-structural analysis and 3D geometric morphometrics. This approach was designed to precisely quantify tool geometry and uncover their functional potentials. The analysis revealed significant variability within the techno-structural groups, often intersecting with typological classifications. These results indicate that despite their production attributes, unifacially shaped artifacts demonstrate considerable morpho-structural diversity. The study delineated nine distinct techno-structural groups, each suggesting potentially different functional organizations and deviating from conventional typologies. These results indicate that unifacially shaped artifacts, while embodying a novel technological paradigm of production, exhibit a broader spectrum of variation mainly due to different tool functions. The combined approach adopted in this research highlights on the cultural significance of unifacial tools within Paleoamerican technological systems. It suggests probable unique tool concepts specific to the study area, challenges existing classifications, and enriches our comprehension of early lithic technology in South America.

## Introduction

The study of lithic artifacts plays a pivotal role in understanding the technological behaviors of early human groups. Traditional typological and metric analyses have long been employed to investigate assemblage variability, spatiotemporal development, and human technological evolution [1–4]. While typological approaches provide valuable insights, they often rely heavily on the analyst’s experience, leading to issues of low reproducibility and classification bias [2]. Traditional metric methods, though more objective, are limited in their focus on size over shape and their inability to fully capture spatial relationships between measurements [2, 5–7].

Geometric morphometrics (GM) provides an effective alternative for quantitatively capturing and preserving shape and form information throughout statistical analysis [8]. Since its adoption in the natural sciences during the second half of the last century, GM has proven to be a valuable set of statistical tools for studying morphological variability within a wide range of research questions at the interface of ecology and evolution [9–12]. Over the past few decades, its application has expanded beyond biological research, including the study of material culture in archaeology [13–16]. GM’s advantages over traditional linear morphometrics have been empirically demonstrated in numerous studies, particularly in analyzing shape- and form-related aspects of tool use, edge resharpening, and technological variability [1, 6, 17–39]. Today, GM analysis of lithic assemblages has outgrown its novelty status, becoming a standard methodological approach in archaeological research [8].

To address these limitations, the present research integrates a methodological framework combining a Techno-structural analysis with 3-D geometric morphometric methods. This dual approach enables a comprehensive examination of lithic artifacts from Central Brazil’s Holocene period (with a lower limit of ~ 7000 BP), focusing on structural variability in unifacial tools associated with the Itaparica technocomplex. By quantitatively capturing tool geometry and interrelating it with technological, functional, and ergonomic factors, this study aims to explore the factors governing variability within these artifacts ‘structure while situating them within broader archaeological and environmental contexts.

The variability observed in unifacially shaped tools from the Itaparica technocomplex is hypothesized to result from a combination of factors, including environmental conditions, raw material constraints, technological strategies, savoir-faire, and socio-cultural practices [40–43]. Additionally, the influence of chronological shifts, shaping techniques, successive transformations (reconfiguration and recycling) and site functions on artifact design and transformation is considered [44–46]. Prior research suggests both heterogeneity and cohesion within Central Brazil’s lithic productions, reflecting the complex interplay of micro-regional techno-cultural specificities and overarching technical systems [47, 48]. This study builds on these perspectives by applying an integrative approach that combines qualitative and quantitative analyses to capture artifact variability at intra- and inter-site levels.

The lithic assemblage analyzed in this research is derived primarily from the GO-NI-01 site (“Caieira Barreiro”) and represents a crucial archaeological collection housed at the Museu Antropológico of the Universidade Federal de Goiás. Situated in Central Brazil, this region encompasses diverse morphoclimatic domains, including the Cerrado and Caatinga formations. These environments are rich in biodiversity and host a dense concentration of prehistoric sites [49, 50]. The GO-NI-01 site provides a critical lens for understanding early Holocene technological and adaptive strategies, particularly in the context of significant environmental and climatic shifts during the Pleistocene-Holocene transition [51, 52].

This study focuses on 67 unifacially shaped artifacts, representing 1.73% of the total lithic collection from the site. These artifacts were selected based on strict criteria, emphasizing their ergonomic attributes, functional potential, and roles within the *chaîne opératoire* of lithic production. The central question driving this research is: What factors govern the variability within and between unifacially shaped tools from the Itaparica technocomplex in Central Brazil? This study hypothesizes that the variability observed is a result of a combination of factors, including raw material availability, shaping methods, functional and ergonomic requirements.

To test this hypothesis, GM offers a transformative alternative to traditional morphometric approaches by quantitatively preserving shape and form through statistical analysis [8]. Unlike conventional methods, GM captures relational shape information using Cartesian coordinates and landmarks, allowing for high-resolution analysis of morphological variability [18]. Semilandmarks, in particular, enhance the capacity to analyze complex shapes by capturing curvatures and surfaces, minimizing biases associated with arbitrary point placement [2].

By integrating GM with Techno-structural analysis, this research goes beyond morphology to examine the structuration, functional potential, and adaptability of these artifacts. This combined methodology addresses gaps in existing studies, which often fail to systematically explore the interplay between technological and cultural factors across different spatial scales.

### The chronological and cultural significance of unifacially shaped artifacts in the first settlement of the South America and Brazil

The emergence and definition of unifacial technology in South America have been a topic of scholarly debate since the 1970s [53–57]. The term “unifacial” itself has been interpreted variably, ranging from early traditions of flake tools with unifacial and peripherical marginal retouch (*Edge-Trimmed Tool Tradition*) [58] to later traditions involving unifacial shaping. In this context, we focus on the latter, particularly associated with the initial dense settlement in northern and central Brazil [59, 60].

Unifacial shaping, a common mode of blank production in South America between the end of the Pleistocene and the mid-Holocene, presents an innovative approach in the deep history of lithic technologies. This technology, as detailed by Lourdeau [41], involves shaping large flakes only on their dorsal face. These pieces, often referred to as “limaces" or “plano-convex tools” are distinct from unifacial retouch flake tools, as they are shaped to alter the initial volume of the blank, not just to create or modify the active parts of natural/initial of the tool morphology.

The Itaparica technocomplex, predominantly observed in central and northeast Brazil, is renowned for these unifacially shaped lithic tools [41, 61], but not defined solely by this type of unifacially shaped artifact. Rather, it represents a comprehensive technical system, of which these objects are merely a component [60]. Detailed analyses, such as those of the Serranópolis collections [59], reveal the multipurpose nature of these tools, with several different functions and independent functional parts on the ends and/or lateral edges, indicating a versatility akin to some bifacial pieces.

The Itaparica technocomplex chronology provides critical insight into early human settlement in Brazil, beginning after 13,000 cal BP. The earliest unifacial tools appear at Lapa do Boquete, Minas Gerais, dated to 14,000–8,500 cal BP [45, 62, 63]. Other Minas Gerais sites, such as Lapa dos Bichos, Lapa do Dragão, and Lapa do Boqueirão Soberbo, date between 13,000 and 9,000 cal BP [63–67]. In southeastern Goiás, Serranópolis shelters show rich unifacial artifact concentrations dating from 12,500 to 9,500 cal BP [48]. Similarly, open-air sites in the Tocantins River valley (e.g., Miracema I, Capivara 5) date to 12,500–10,000 cal BP [68]. Mato Grosso and Mato Grosso do Sul sites, including Santa Elina, span 12,500–8,000 cal BP, with Santa Elina providing recent examples within this range [69–71].

In Serra da Capivara, northeastern Brazil, sites such as Toca do Boqueirão da Pedra Furada, Toca do Sítio do Meio, and Toca da Cerca do Elias, among others, date from 14,000 to 8,000 cal BP and exhibit unifacial tool production [59].

In contrast, southern Minas Gerais sites like Lagoa Santa lack unifacial tools, focusing instead on quartz blocks and crystals reduction using unipolar and bipolar-on-anvil techniques, producing minimally retouched flakes (12,000–9,000 cal BP) [72–78]. Santana do Riacho and Lapa Pequena reflect similar quartz reduction patterns [79–81].

The "Itaparica tradition," defined by Calderón [82] and Martin [83], originated as a local group but evolved into a broad cultural unit across central and northeastern Brazil during the Pleistocene-Holocene transition and the early Holocene. Characterized by unifacial tools, this tradition reflects subsistence strategies in the tropical savannah [57, 84, 85]. These patterns highlight regional variations in lithic production, emphasizing diverse strategies among Brazil’s early inhabitants.

However, the nomenclature and definitions of the Itaparica tradition and its associated lithic material have been subject to considerable variability and subjectivity across different authors. This disparity in definitions, highlighted by the diverse terminologies used to describe unifacially shaped artifacts (“lesmas”, “limace”, “rabots", “semi-circular plano-convex scrapers”, etc.), indicates a relative subjectivity in the archaeological understanding of this cultural assemblage. Amidst this terminological diversity, two main definitions of unifacially shaped artifacts stand out as cultural markers since the 1970s due to their distinct morphology: the morpho-typological definition of “limace” (“lesma”), and the techno-structural definition by Lourdeau [41]. Bordes [86] defines “limace" as a tool with typically blunted ends, often featuring complete steep retouch, and occasionally retaining a cortex on the dorsal face. Lourdeau [41], on the other hand, offers a techno-structural definition, introducing the term “*Pièce Façonnée Unifacialement à une Face Plane*” (PFUFP), highlighting a principle of shaping large flakes unifacially on the upper face while keeping the lower face flat, and suggesting a multi-functional potential. The PFUFP concept, as characterized by Lourdeau [41], is defined by several key principles:

- Volume and Shape: PFUFPs are typically elongated and moderately thick with one flat face. This shape is a result of unifacial shaping on large flakes. In general, the symmetric profile 1 (see later Fig 5) has a length ranging from 4.5 to 15 cm, associated with a thickness between 1 and 4.5 cm. The symmetric profile 2 and asymmetric profiles are linked to lengths between 4.5 and 11 cm and thicknesses between 2 and 4.5 cm.
- Production Principle: These tools are created from large flakes, shaped unifacially on the upper face while the lower face remains flat. This method distinguishes PFUFPs from other lithic tools. The flakes used for the production of PFUF with symmetric profile 1 mainly have a width between 2 and 7 cm. As for the PFUF with symmetric profile 2 and asymmetric profiles, the selected widths vary between 2 and 6 cm.
- Functional Principle: PFUFPs are designed to support at least one transformative techno-functional unit at the apical end, often with an axial grip at the base. It is probable that these tools were also designed for attachment to a handle.
- Longevity: The structure and material reserve of PFUFPs allow for multiple phases of sharpening and reshaping before they are exhausted, indicating a significant lifespan.

In summary, PFUFPs as described by Lourdeau are notable for their distinctive volumetric shape, production methods, multifunctional potential, and durability. However, as remarked also by Lourdeau [41], this concept of PFUFP reflects also a notable variability, in several aspects:

- Raw Material Diversity and its implication: In central Brazil, the raw materials used for PFUFPs include sandstone, flint, and quartzite, suggesting that the specific type of raw material is not a crucial factor in their production [87, 88] contrary to what was observed by Bueno et al. [49] in Tocantins.
- Volume and Cross-Sectional Variability: PFUFPs demonstrate a range of profiles and cross-sections, reflecting variability in volumetric properties. This includes different profiles like trapezoidal, semi-circular, and triangular cross-sections, each achieved through specific flaking and shaping methods relative to the target final volume. Technological and structural research has revealed that the methods employed in flaking and shaping are closely tied to the target volume and type of cross-section [41].
- Techno-Functional Variability: Each PFUFP features transformative techno-functional units with varied delineations (rounded, pointed, or straight transversal). The diversity in base planes, penetration planes, and contact planes indicates variations in the functional potentials of the tools.
- Multiplicity of Tools: Beyond the primary axial tool present in all PFUFPs, these artifacts sometimes contain additional transformative units, numbering up to three. These are located on one or both sides or on the end opposite the primary transformative unit. The volumetric and techno-functional variability has led to the differentiation of PFUFPs into two major categories: PFUFP tools, designed to contain only one tool, and PFUFP tool-supports, which serve as matrices for several tools.
- Reconfiguration Patterns: The reconfiguration or reshaping schemes of PFUFPs add another layer of variability, independent of their volumetric and functional diversity.
- In summary, the concept of PFUFP as explored by technological analysis highlights a remarkable degree of variability in production methods, materials used, functional potential, and structural design. These artifacts not only represent a unique lithic technology but also indicate a diversification in tool-making strategies during the Pleistocene-Holocene transition and the early Holocene. Finally, unifacial artifacts are found in Brazil and other parts of America, both within and outside the Pleistocene-Holocene transition, challenge the concept of a "type fossil" for the Itaparica phenomenon. These artifacts, similar to those in the Itaparica Technocomplex, are part of different technical systems [60]. This includes Amazonian sites with bifacial components [89–92] and southern Brazilian sites with predominantly bifacial industries [60]. Recent Holocene unifacial artifacts differ structurally from earlier ones, suggesting independent development rather than a historical-cultural link [60]. The Itaparica Technocomplex itself also includes unique asymmetrical unifacial pieces, especially from its early phase in Serra da Capivara [93, 94] and the Santa Elina site [70].

Considering this, the presence and characteristics of these unifacially shaped artifacts across different regions and time periods in South America not only emphasize their importance in the prehistoric toolkit but also suggest dynamic technological strategies. The variability in structure and usage, particularly in the Itaparica Technocomplex, reflects a nuanced and adaptive approach to tool-making that was not confined to a single period or region. These findings open up new reflections for understanding the technological diversity and innovation among early human populations in South America, inviting further research into the complexities of their lithic strategies, their environmental and cultural implications.

### Different ways to approach the cultural significance of unifacially shaped artifacts of the Itaparica technocomplex

Exploring the cultural significance of unifacially shaped artifacts requires diverse analytical perspectives, combining morpho-typological, technological, structural approaches, and use-wear studies. While technological studies have primarily focused on defining industries at certain sites in central and northeast Brazil [45, 87, 95], a more comprehensive analysis incorporating the entire production system has been lacking. Recent works by Lourdeau [41] and other researchers in Brazil [43, 47, 94, 96–100] have initiated this broader technological and structural analysis of unifacially shaped artifacts. These efforts aim to bridge the functional and cultural dimensions of these tools, acknowledging that their roles may extend beyond purely utilitarian purposes to reflect broader cultural characteristics and practices.

The traditional morpho-typological approach, which considers the final silhouette of the tool, has been supplemented by a technological perspective focusing on the entire production process, including the different reduction sequences. This is complemented by a structural approach that considers each tool as comprised of three universal parts: a transformative part, a prehensile part, and an intermediate energy-transmitting part [101, 102]. These approaches have confirmed the conceptual homogeneity of unifacially shaped artifacts of the Itaparica Technocomplex at certain Brazilian sites, while also revealing significant morphological, volumetric, and functional variability [103].

Additionally, reduction sequences for these artifacts have shown considerable variability from one site to another, with different cycles of support production, reconfiguration, and recycling [45, 46]. This indicates a cultural and technological complexity that goes beyond narratives focused solely on “plano-convex” tools [103]. For instance, in the Diamantina region of Minas Gerais, unifacially shaped artifacts dating to the late Holocene (post 2000 BP) were found to be similar to those from the Pleistocene-Holocene transition and early Holocene in other areas of central Brazil, yet with distinct archaeological contexts, manufacturing processes, and blanks, in this case slabs [103].

From a techno-structural perspective and the technological organization approach, the various phases of reconfiguration identified in the study of these unifacially shaped artifacts from different regions suggest that their formal diversity results significantly from operations of reconfiguration, including significant volumetric restructurings [41, 44, 45]. These reconfigurations can be understood through concepts like homothety, where proportional scaling of artifact dimensions occurs, as well as through allometry, where changes in the relationships between different parts of the artifact emerge over time. Applying these concepts to archaeology, such transformations resonate with the Frison Effect [104] and foundational works by Jelinek [105] and Dibble [106–108], and other more recent studies [109, 110] which emphasize the dynamic morphological evolution of tools during use and reconfiguration.

This view is supported by raw material procurement strategies, with a clear selection of the best-suited varieties (fine-grained) for producing plano-convex artifacts, observed across various sites [40, 41, 63, 59, 93]. Studies from Vale do Peruaçu and Serranopolis (GO-JA-01) [45, 46, 95] demonstrate how significant morphological transformations throughout the lifecycle of these artifacts can lead to a misreading of formal diversity, where different stages of reconfiguration may be mistakenly categorized as distinct types. Acknowledging these transformations as allometric shifts offers a more nuanced understanding of artifact variability.

Furthermore, the practice of reconfiguration suggests that these pieces were crafted for longevity rather than single-use followed by discard. Bueno highlights their multifunctionality and capacity for reconfiguration, framing this as part of a techno-economic strategy aligned with the concept of “transportability”. This provides dynamic, spatial, and territorial guidelines, emphasizing the adaptability and extended utility of these artifacts [40].

However, a finer understanding of the shape variability of these artifacts is needed in order to examinate, in a mixed quantitative and qualitative manner, factors such as site function, tool function, technological context, raw materials, blank types, reconfiguration schemas, shaping modalities, and location of active or prehensive parts. 3D geometric morphometric methods (GMMs) combined with a techno-structural perspective, holds the potential for mediate this type of research. More broadly, in others parts of Brazil 2D morphometrics had been successfully applied to Paleoamerican lithic technology [111, 112]. The use of 3D models can facilitate and objectify comparisons among researchers working on shared digital models, and allows to examine large assemblages of items using advanced statistical methods as has already been demonstrated in other contexts [18, 19, 21, 25, 37, 113–117]. In summary, the cultural value of unifacially shaped artifacts must be questioned through multiple analytical frameworks, including typology, technology, structural analysis, and traceology. These combined approaches should allow for a more comprehensive exploration of the tool’s place within the mental templates of the first Brazilian human groups. Particularly, the morpho-volumetric objectives behind their production should be considered in tandem with technological information, as a complement to understanding their functional role.

## Study area: The case of GO-NI-01

**Fig 1:**
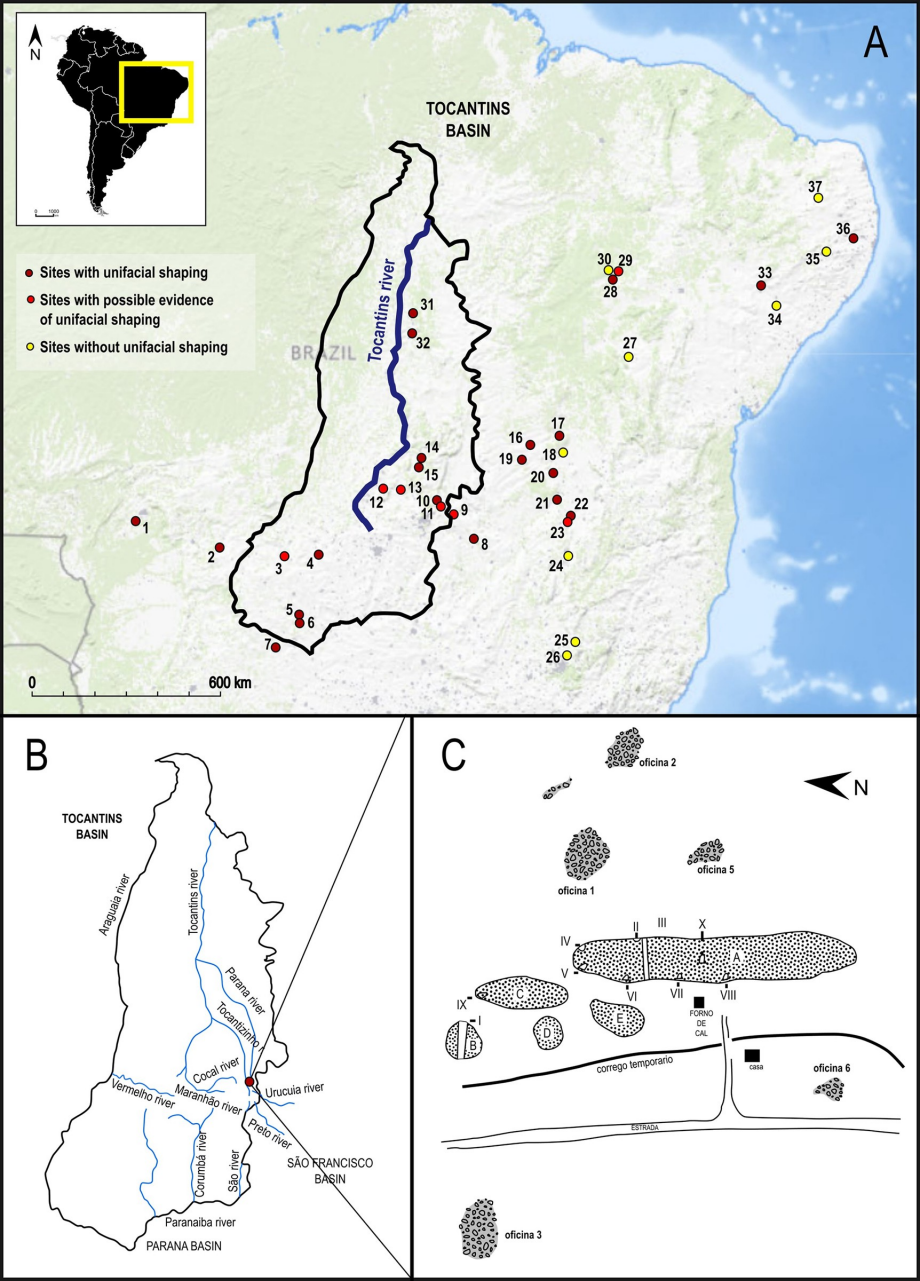
Geographic location of the study area. Map in A obtained from the USGS National Map Viewer. Credit: U.S. Geological Survey. (A) Location of the main sites prior to 7,000 BP in central and northeast Brazil and distribution of lithic industries with and without unifacial shaping (numbers refer to site names listed in S1 Table) according to Lourdeau [59, 41]; (B) focus on the Tocantins basin and the Cocal river; (C) Sketch of the GO-NI-01 complex and its various lithic “workshops”, modified from Simonsen [118].

The study area, situated within the broader context of South American and Brazilian archaeology, encompasses a region of central and northeast Brazil known for its rich prehistoric legacy. This region, as illustrated in the geographic location map of the study area (Fig 1A), highlights the main sites dating prior to 7000 BP, with a specific focus on the distribution of lithic industries, both with and without unifacial shaping, as categorized by Lourdeau [41, 59]. Particularly, the Tocantins basin and the Cocal river (Fig 1B) represent key areas in this study, featuring a concentration of significant archaeological sites. Among these, the GO-NI-01 complex, also known as “Caieira Barreiro”, stands out with its diverse lithic “workshops” (Fig 1C), documented by Simonsen [118]. This area serves as a vital link in understanding the early human occupation and lithic technology development in South America, reflecting a unique intersection of cultural, ecological, and technological influences that have shaped the prehistoric landscape of central Brazil.

The archaeological site GO-NI-01 (or “Caieira Barreiro”) in Planaltina, central Brazil, forms a part of a larger ensemble of six open-air sites within the Rio Cocal basin, closely associated with the Bambui formation and the Tocantins river basin. Systematically prospected and excavated in 1974 by the setor de Arqueologia do Museu Antropologico da Universidade Federal de Goiás (MA-UFG), these sites have provided a significant collection of lithic pieces. Among these, 10,026 lithic artifacts were collected, with a majority comprising flakes and used flakes. The tools, predominantly made of flint and chalcedony, feature a notable presence of limaces (10.68% of the material) and rare bifacial projectile points, indicative of the “Cocal Typology” characterized by unifacially shaped plano-convex artifacts [118].

The archaeological site of Caieira Barreiro (Barreiro Limekiln) is located on Fazenda Barreiro, approximately 45 km from Brasilinha, and includes a limestone formation about 20 meters high and 800 meters long, from which raw materials for lime production are extracted. This area encompasses several caves, of which ten have been researched.

The “Cocal Typology” [118] includes 9 lithic workshops and 25 caves in the Bambui limestone formations of eastern Goiás. The lithic industry features bifacial, plano-convex artifacts, typically ellipsoidal, made mainly of silexite/chalcedony, with some quartzite, limestone, and slate. Tools like limaces (17.20%), scrapers, drill-scrapers, axe blades, and throwing points were primarily percussion-made. Artifacts are polyvalent, with use determined by dimensions and retouching rather than morphology.

In the site GO-NI-08 (or “Barreiro”) in Planaltina, an open-air site excavated in 1977 [119], lithic material similar to the two previously mentioned sites was found. This site, primarily featuring flint and quartzite artifacts, yielded a ^14^C date of 10,605 ± 125 BP from charcoal in layer 5, 60 cm deep, in association with lithic remains. Only two unifacially shaped artifacts were recorded at GO-NI-08, and only one was available for this study. A recent PhD dissertation by Betarello [120] demonstrated the presence of four main types of tool-making objectives: multifunctionnal plano-convex tools, retouched blanks, and evidence of bifacial shaping. The stone knapping was carried out solely through direct percussion with hard mineral or organic hammer techniques. The plano-convex tools were mainly made of local siltstone and flint. Finally, cores are rare. In some cases, stationary cores are presents. Others show an orthogonal bidirectional flaking method.

**Fig 2:**
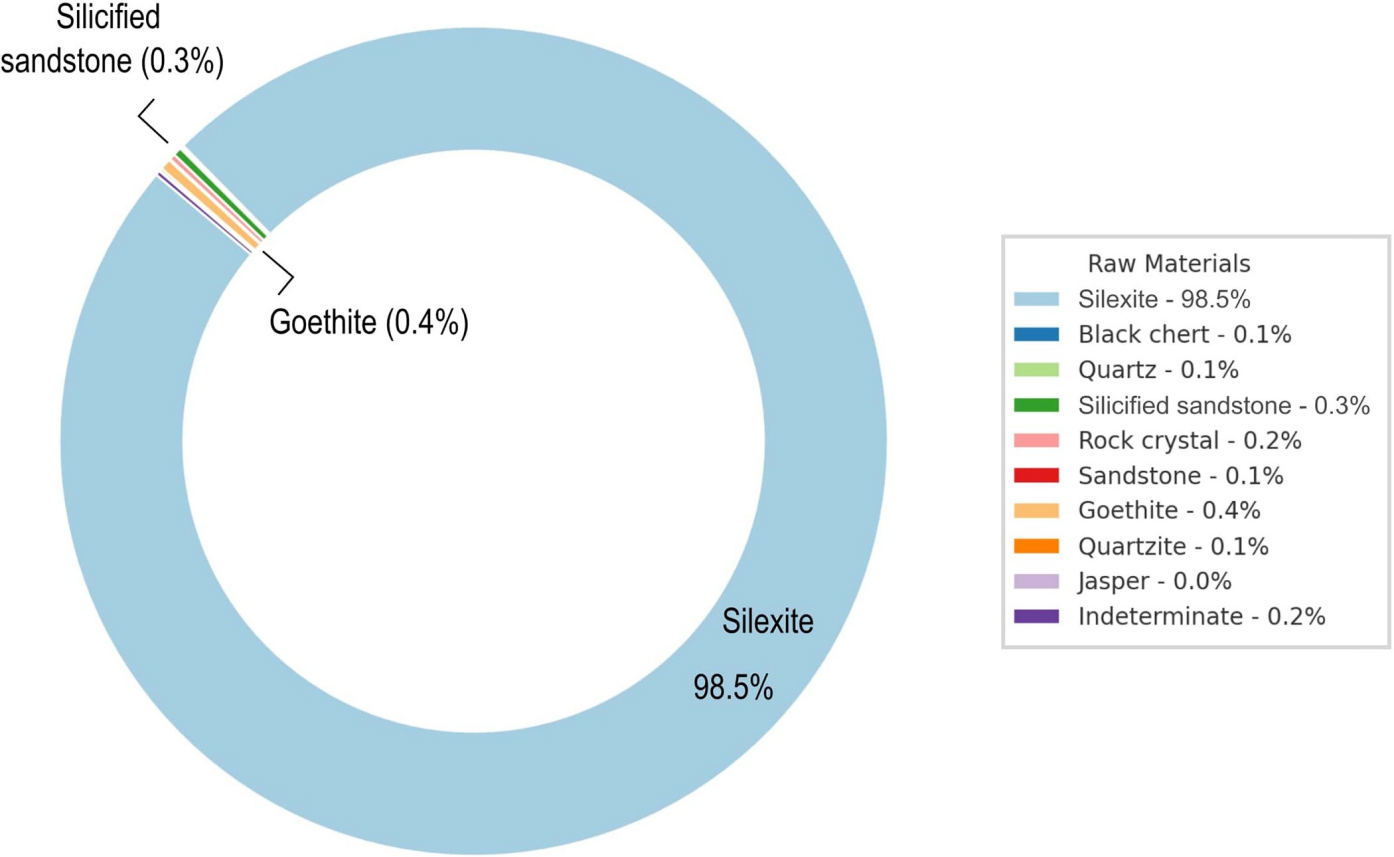
Percentage distribution of raw materials of the GO-NI-01 industry.

The analysis of the 67 pieces carried out in this study forms part of a comprehensive investigation into the chaînes opératoires identified at the sites under consideration. To this end, a total of 3,879 artifacts (3,110 pieces from workshop 1, 918 pieces from workshop 3), each classified into distinct categories based on their technological attributes (Table 1). The selection of these pieces corresponds to the fact that these two workshops present a more significant variability of unifacially shaped tools. 98.5% of this material is manufactured in silexite/chalcedony (Fig 2). Among the total population, unifacial shaping flake emerges as the most prevalent category, accounting for 15.42% of the total count, closely followed by shaping flakes at 14.59%. Notably, miscellaneous fragment forms a significant portion, representing 18.44% of the assemblage. On the other end of the spectrum, several categories such as fragmented elongated flake, fragmented tool on elongated flake, plunging flake *(outrepassé)*, and others, are scarcely represented, each constituting merely 0.03% of the total. Other elements, ranging from *débordant* flakes (0.18%) to wider than long flake (0.26%), offers a comprehensive overview of the production systems within the GO-NI-01 industry, revealing indices of orthogonal and discoidal production systems. Drawing from these findings, we can infer at least five distinct reduction sequences: bifacial shaping (encompassing both projectile points and bifacial tools and unifacial shaping (flake tools, pebble tools, and slab tools). Moreover, the identification of tools on reconfiguration flakes from unifacial artifacts, as well as on unifacial shaping flakes, suggests a complex process involving recycling and confection. These tools on flakes, or micro-tools, can be categorized into unretouched rough tools or into more specific types such as scrapers, micro-denticulates, and notches. More rarely, these tools can be identified as rostrums or becs.

**Table 1:** Counts and proportion of technological categories represented in the GO-NI-01 industry.

	Technological categories	n	%
**Production**	Bifacial shaping flake	28	0,72
	Casson	141	3,64
	Core	15	0,39
	Core waste	9	0,23
	Cortical flake	4	0,10
	*Débordant* flake	7	0,18
	Debris	26	0,67
	Elongated flake	85	2,19
	Elongated shaping flake	8	0,21
	Flake	124	3,20
	Fragmented flake	245	6,32
	Fragmented shaping flake	309	7,97
	Full flaking flake	49	1,26
	Hammerstones	4	0,10
	Initial flaking flake	4	0,10
	Initial shaping flake	2	0,05
	Manuport	35	0,90
	Miscellaneous fragment	715	18,44
	Oblique flake	5	0,13
	*Outrepassé* flake	1	0,03
	Pebble	4	0,10
	Resharpening flake	146	3,76
	Retouching flake	10	0,26
	Semi-cortical	21	0,54
	Shaping flake	566	14,59
	Fragmented elongated flake	1	0,03
	Unifacial shaping flake	598	15,42
	Wider than long flake	10	0,26
**Tools**	Flake tools	410	10,57
	Fragmented bifacial piece	11	0,28
	Fragmented tool on elongated flake	1	0,03
	Fragmented unifacial shaped artefact	150	3,87
	Fragmented tool	1	0,03
	Unifacially shaped artifacts	67	1,73
	Unifacially shaped artifacts reconfiguration flake	47	1,21
	Point	1	0,03
	Slab tools	4	0,10
	Splinter	5	0,13
	Unifacial tools	8	0,21
	Undetermined	1	0,03
		**3879**	**100**

## Materials: Sample composition

**Fig 3:**
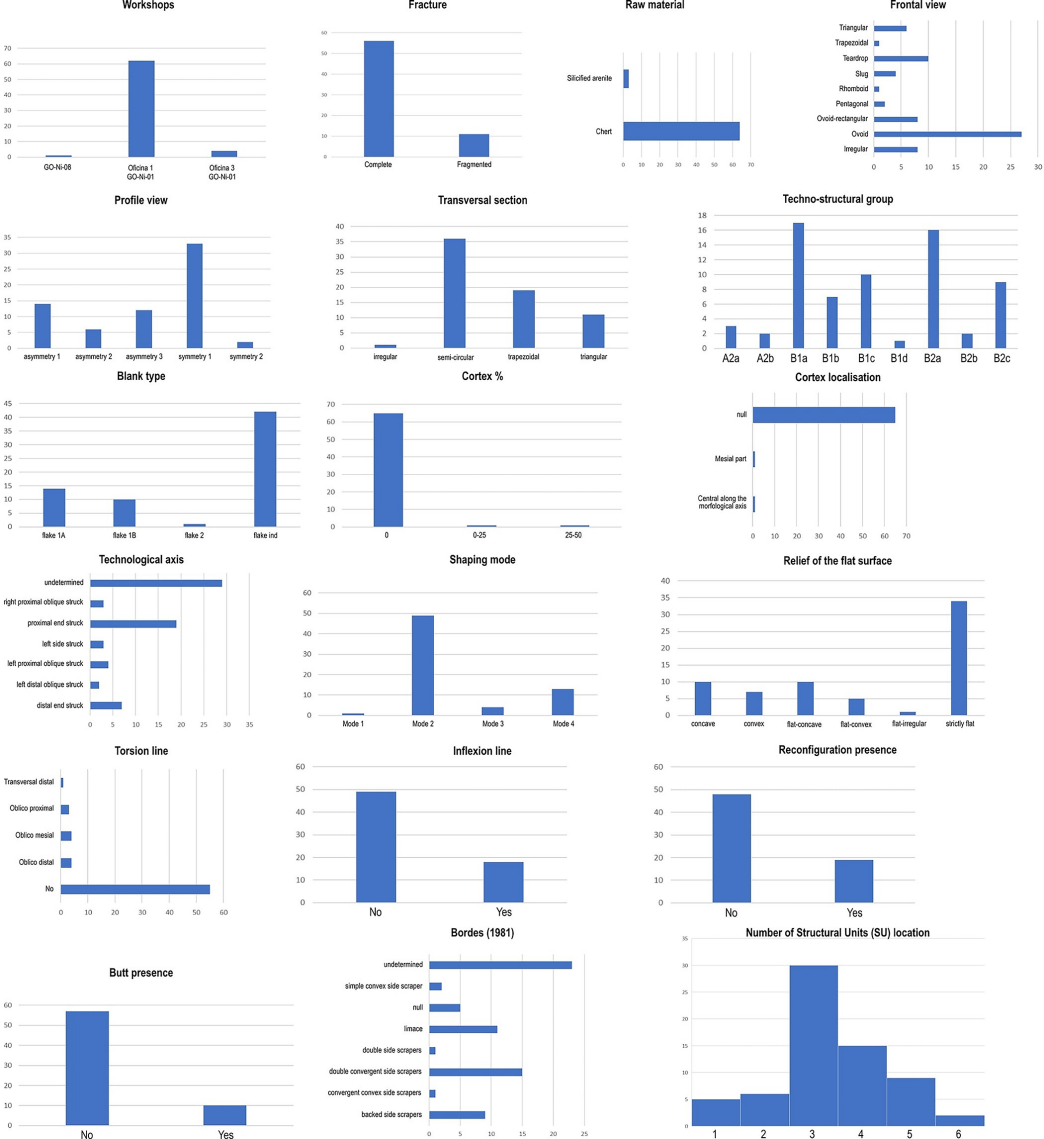
Principal features of unifacial artifacts sample (n = 67) of this study according to qualitative and quantitative criteria.

This study concentrates on 67 unifacially shaped artifacts, representing 1.73% of the total collection technologically studied and stored at the *Museu Antropológico (MA) of the Universidade Federal de Goiás (UFG)* in Goiânia, central Brazil. The totality of these artifacts, except for one, originate from the GO-NI-01 site. As previously mentioned, the remaining piece is from GO-NI-08, included in this study as it is one of the two “limaces” reported at this site, with the other being unavailable for analysis at the time of this research (Fig 3). The manual 3D analysis database can be found in S2 Table.

The selection of these specific artifacts was based on several criteria: (1) their ergonomic characteristics and seemingly simple and homogeneous functional potentials: presumably clearly identifiable transformative and prehensile parts suggesting a limited range of functional possibilities, (2) their representation as key elements in the mixed *chaînes opératoires* of flaking and shaping, and (3) their exemplification of a clear rupture between periods before and after their emergence in the archaeological record. Although part of the reasons for selecting the GO-NI-01 site were pragmatic (e.g., access to the collection), it was primarily chosen due to its significant number of well-preserved unifacially shaped tools, including nearly the entire reduction sequence of their production. This site offered good conditions for analyzing the variability within and between these tools, using both manual structural technology and automated 3D geometric morphometrics, as the tools exhibited minimal fractures and reconfiguration.

**Fig 4:**
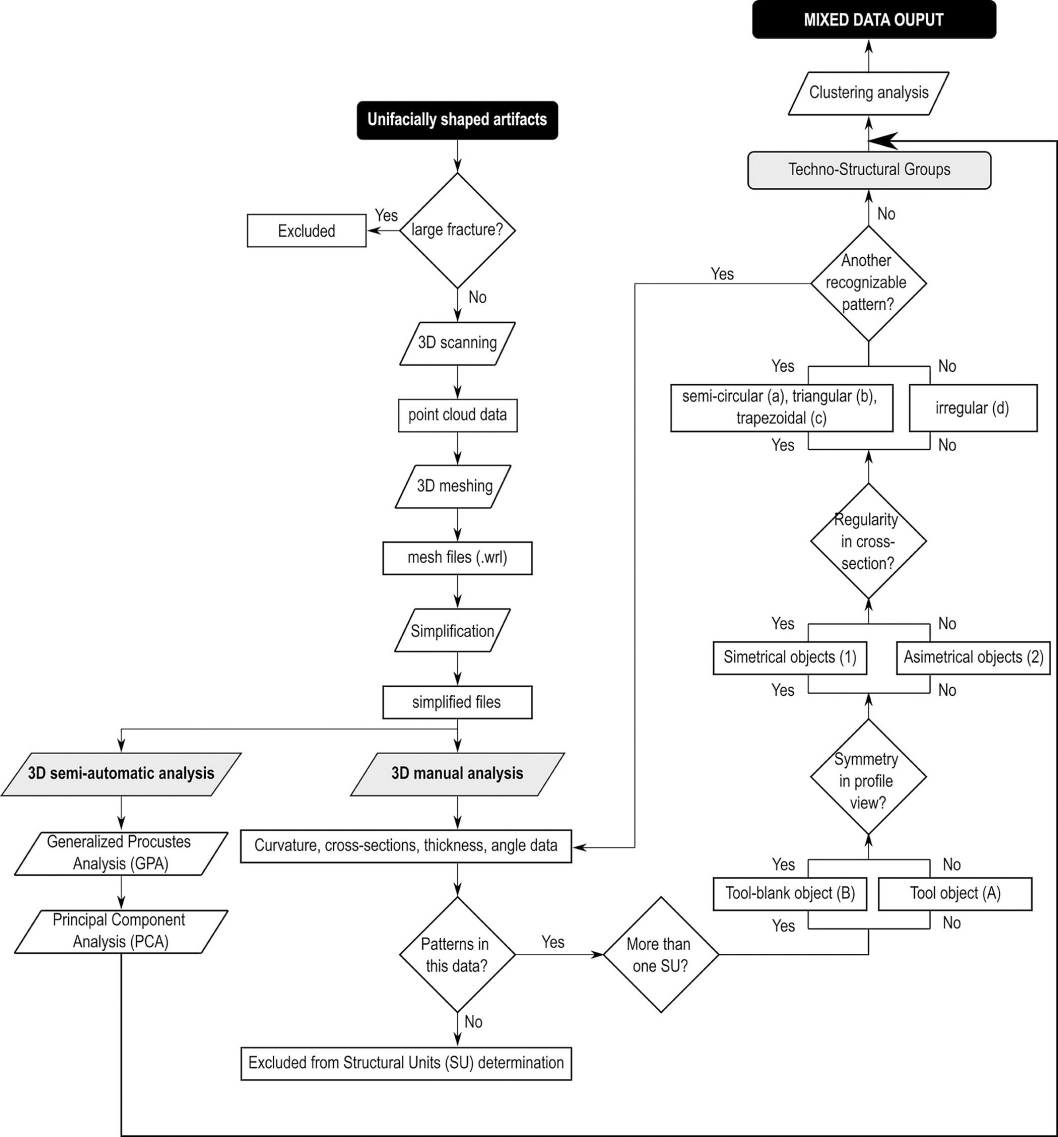
Flowchart of classification of “techno-structural” groups in this study.

A flowchart, as shown in Fig 4, outlines the decision-making process that led to the composition of the sample. The primary criterion for selection was the absence of large fractures that might disrupt the original delineation of the pieces. In one unique case, a fragment of a corresponding piece was located, allowing for a virtual refit. The chosen sample thus facilitates a comprehensive analysis, offering both quantitative and qualitative comparison opportunities. This selection also provides a complete overview of the techno-structural variability of the GO-NI-01 occupations, allowing for an in-depth understanding of the artifacts’ characteristics and their implications in the broader archaeological context of central Brazil.

## Methods: 3D geometric morphometrics and techno-structural analysis

Our study applicated a combined approach between 3D geometric morphometry semi-automatic analysis and techno-structural manual analysis (Fig 4) in analyzing GO-NI assemblages from central Brazil, aiming at research the factors governing their variability.

Before conducting the combined analysis, we carried out a taphonomic assessment of the sample, evaluating surface preservation, ridge and edge abrasion, patinas, crushing, texture, and coloration. Following this, we analyzed the metrical characteristics of the tools using descriptive statistics, including means, ranges, and standard deviations for length, width, and thickness. To examine dimensional relationships and test for allometry, we conducted regression analyses. Kruskal-Wallis tests were employed to assess differences in medians across techno-structural groups, while Mann-Whitney U tests were used for pairwise comparisons, with p-values adjusted to control for Type I errors. These statistical methods aimed to identify variability patterns and explore potential relationships among metrical attributes within and across tool groups.

### Techno-structural analysis

**Fig 5:**
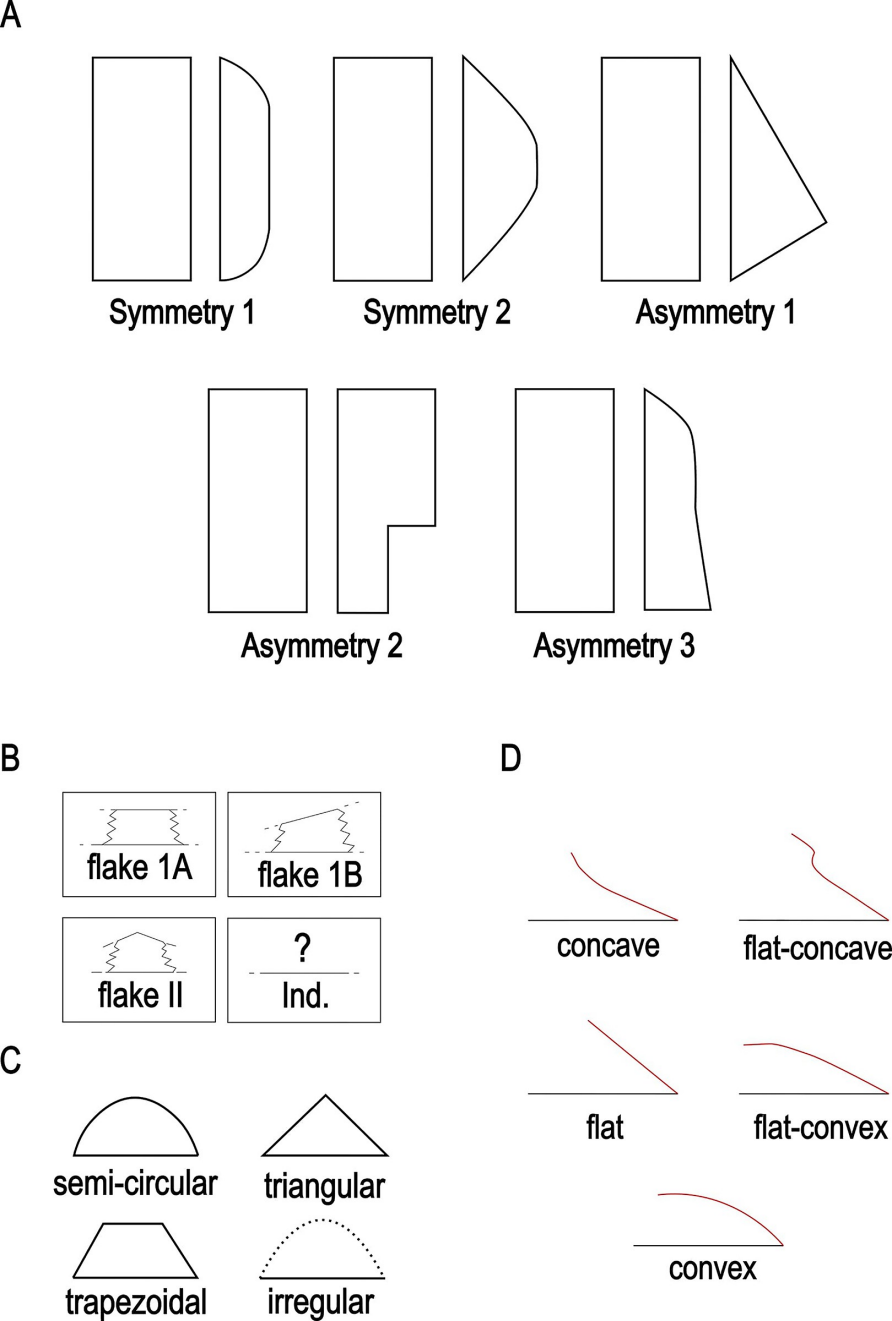
Categories of symmetries (A), blank type (B), transversal sections (C) and cutting-edge bevel in (D) cross-section view considered in this analysis (modified from (61), p. 101, Fig 25, and [25], 2020, p. 14, Fig 4).

**Fig 6:**
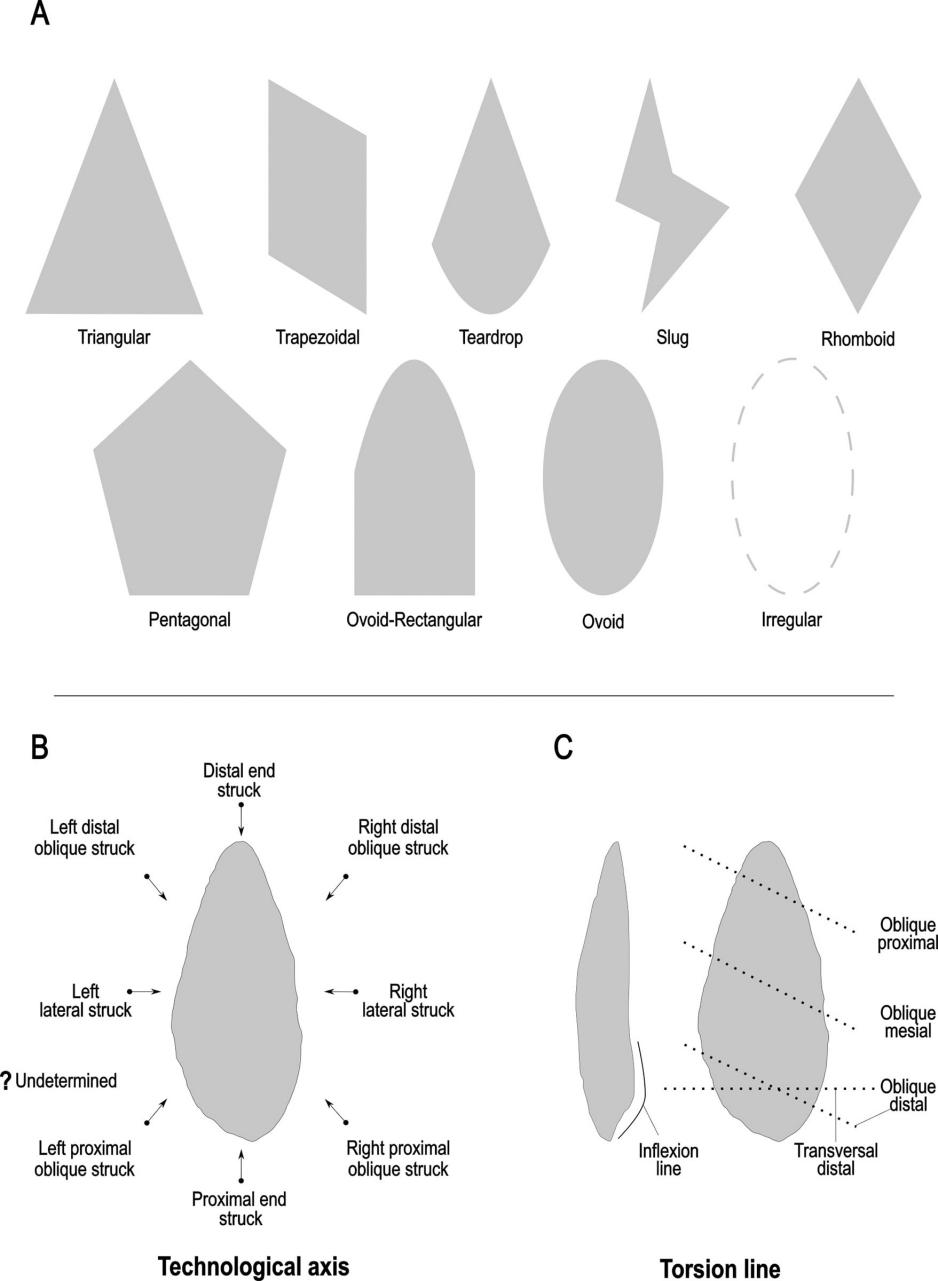
**Details of the qualitative criteria considered in the analysis.** (A) Graphic representations of Frontal view, (B) types of technological axis, (C) types of torsion lines and inflexion line.

**Fig 7:**
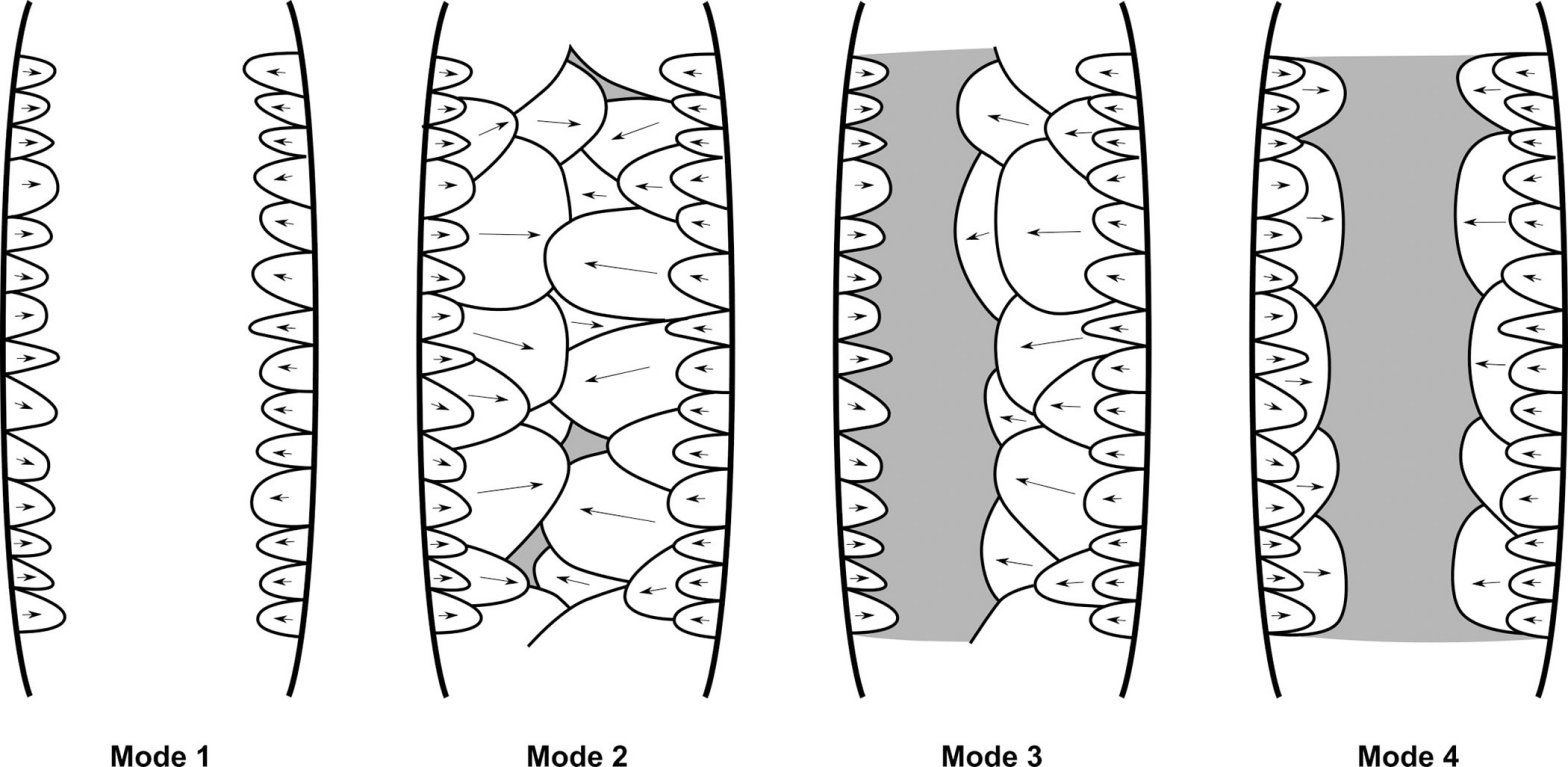
Four modes of shaping considered in this analysis (modified from [41], p. 148, Fig 63).

The techno-structural analysis views tools as means of energy transfer, focusing on their techno-functional units (TFUs): prehensive, receptive, and transformative units [101, 102, 121]. This perspective considers the entire lifecycle of a tool, from planning and raw material procurement to use and abandonment. Contrary to classical approaches, this study refrains from interpreting the functional potential of edges, considering the TFUs as structural rather than functional units [121, 122]. The tools are categorized into techno-structural groups without assigning *a priori* specific functional or production interpretations. This methodology aims to identify recurrent technological and morpho-functional features, summarizing them into techno-structural groups. Such grouping is facilitated by a systemic approach that structures and relates various elements, reflecting a level of organization that allows investigating general structural, technological, and ergonomic aspects of the tools [25]. In contrast to the classical techno-functional approach defined by Lepot in 1993, this study does not interpret the functional potential of the edges of unifacially shaped tools. The concepts of prehensive, receptive, and transformative units are deemed too speculative, especially for tools with open-angled edges, as highlighted by Capdevielle & Colongo [122], and Viallet [123, 124]. This perspective challenges the ability to definitively assign a productive or functional purpose based on the presence of negatives of removals, which might also indicate flake production. Instead, this study focuses on grouping artifacts with similar functional or productive potential, which would have been used similarly in Paleoamerican toolkits. Here, techno-functional units (TFUs) are redefined as structural units, leading to the classification of tools into “techno-structural” groups. These groups do not assign *a priori* functional or production interpretations but focus on structural organization. The methodology employed aims to identify and categorize tools based on recurring technological and morpho-functional characteristics (Figs 5–7). This process involves a systematic approach that organizes and correlates different elements of the tools, leading to the formation of tool systems.

Data for attribute and morphometric analysis were collected through both 3D scans and manual recording, following the protocol developed by Pérez-Balarezo & González-Varas [125]. This includes cortex amount, weight, blank type, general shape, Scar Density Index (SDI) *sensu* Clarkson [126] and various other technological features [127] (Figs 5–7).

Four types of flake blanks are distinguished: Type I.A., Type I.B, Type II, and indeterminate (Fig 5). The first type, I.A., involves the remaining scars creating a flat central surface parallel to the bottom face. Type I.B is characterized by the remaining negatives creating an oblique flat central surface in relation to the bottom face. Type II is defined by the remaining negatives creating two surfaces slanting in relation to the bottom face, the intersection of which forms a longitudinal ridge in the middle of the flake. And, the last type of flake-blank, flake ind., does not contain elements that can determine its nature.

Four modes of shaping are distinguished in these pieces (Fig 7). Mode 1 involves short extractions focused along the edge and bevel of the artifact. Mode 2’s shaping phase includes a series of sequential extractions performed on covered sections opposite each other, removed from both sides. The pattern of these extractions often mirrors what’s described as a “candelabra” formation, aiming to smooth out surfaces by minimizing the ridges dividing two such extractions [128]. The impact of these shaping processes is critical, leading to notable changes, or even a complete transformation, in the top surface’s appearance of the base material [41]. In Mode 3, short extractions are made on one side, whereas the other side receives treatment akin to that of Mode 1 artifacts, featuring consecutive sequences of covering extractions in a ’candelabra’ arrangement [41]. Mode 4 is characterized by substantial side extractions but lacks the ’candelabra’ configuration observed in Modes 2 and 3, thus not markedly transforming the blank’s upper surface.

**Fig 8:**
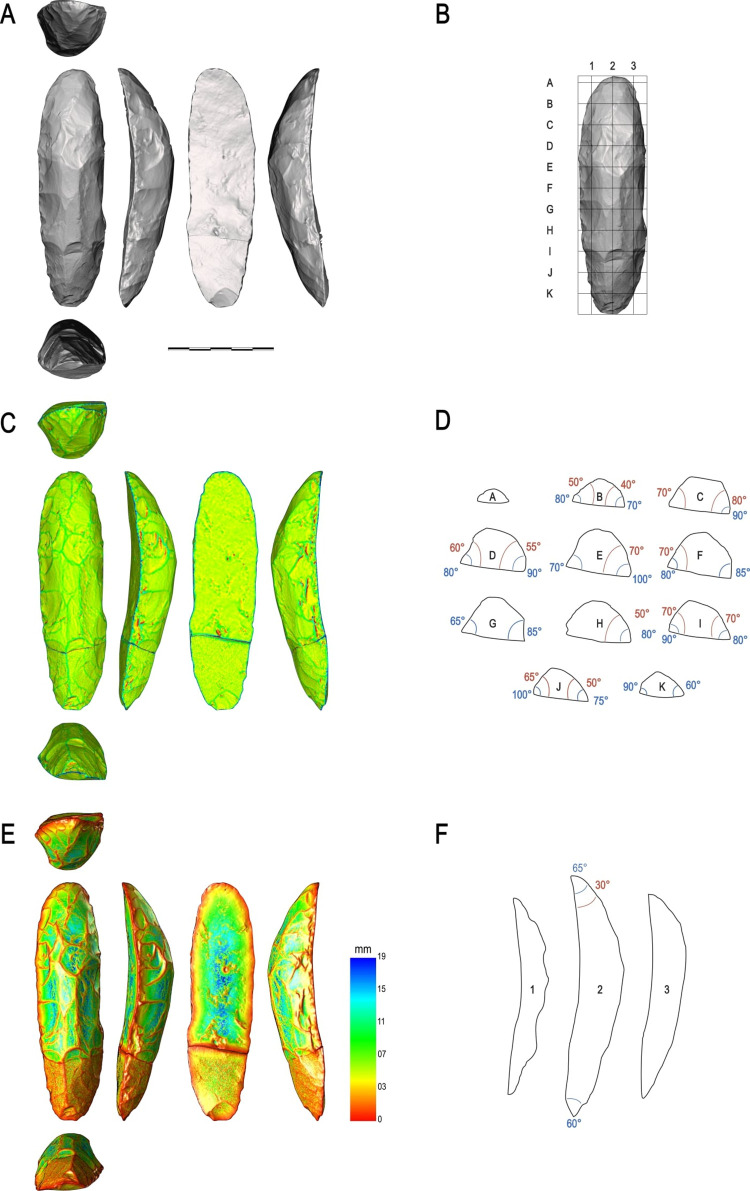
Example of manual 3D analysis of a unifacially shaped tool in this study. (A) mesh model; (B) Systematic gridding, each grid is 10 mm^2^; (C) taphonomical analysis; (D) transversal sections and edge angle measurements; I thickness analysis; (F) longitudinal sections and edge angle measurements.

Our protocol facilitated the identification and analysis of Structural Units (Sus) based on these criteria. The Structural Unit (SU), similar to the Techno-Functional Unit, encompasses a set of comparable technical criteria without making initial assumptions about the artifact’s technical functions (whether transformative or prehensive). These SU are defined by areas of the pieces that exhibit a certain recurrence of morpho-technical elements related to edge geometry such as similar angles, similar edge delineations, and similar sections. Its objective is to diminish the subjectivity in interpreting the technical object by systematizing the technical criteria employed in its analysis (Fig 3). The interpretation of these functions will be conducted subsequently, during the synthesis of Structural Units across all tool groups identified within the lithic collection. At this juncture, Structural Units will be defined as either transformative or prehensive based on their interaction with the corresponding 3D volume. See Fig 8 for an example of this manual procedure.

This protocol incorporates stages like data acquisition, meshing, post-processing, and analysis. It utilizes advanced technologies such as the Shining 3D Scanner–EinScan SP V2, ensuring precision in capturing the details of the artifacts. The protocol covers various analyses including taphonomic, relief, cross-sections, and center of mass determination. The focus is on precise morpho-metrical measurements, and the models are processed for computerized morphometric and statistical analysis [24, 124, 129–132].

The 3D models allow dissecting the tool into parts and investigating spatial relationships between selected points. Tools are aligned along the main axis for accurate measurements and analysis. Sections obtained every 10 mm, both transversal and longitudinal, enable a detailed visualization of the tool’s aspects, like the cutting edge angle and the relationship between possible active and passive parts (Fig 8).

The next step was to conduct a manual classification to form techno-structural groups, i.e., groups of pieces defined according to the following steps (Fig 4):

1. Do the pieces have more than one Structural Unit (SU)? If the answer is yes, they were assigned to group B. If the answer is no, they were assigned to group A.
2. Are the pieces symmetrical in profile view? If the answer is yes, they were assigned to group 1. If the answer is no, they were assigned to group 2.
3. Do the pieces have regularity in the cross-section? If the answer is yes, the type of cross-section was determined. If it was a circular section, the letter “a” was assigned; if it was a triangular section, the letter “b” was assigned; if it was a trapezoidal section, the letter “c” was assigned. If, on the other hand, the cross-section was irregular, the letter “d” was assigned.

In this study, only these three criteria were the common denominators across the entire sample studied. Other criteria, such as the delineation of the SU in frontal and profile view, did not prove to be diagnostic enough to include them as structural classification criteria. The criteria outlined above have been commonly used in manual technological analyses in various South American contexts and have proven useful in defining groups of pieces that aim to address the geometric structuring of unifacially shaped artifacts.

### 3D geometric morphometry

To investigate shape variability in lithic tools, we conducted a geometric morphometric analysis on 3D models using the AGMT-3D software [20], a method increasingly applied in archaeological studies, especially for bifacial tools [19, 24, 133]. For this analysis, a grid of 50 meridians and parallels was applied to generate 2,500 semi-landmarks per surface, capturing detailed morphological data. Generalized Procrustes Analysis (GPA) was employed to normalize the location, orientation, and scale of the models. This statistical technique ensures that comparisons focus solely on shape by eliminating differences caused by size, initial positioning, or scanning methods. Without GPA, such variations could distort the analysis. Principal Components Analysis (PCA) was then applied to quantify shape variability, visualized through scatterplots of the first two principal components.

In the PCA scatterplots, artifacts were color-coded by typological and technological attributes, with confidence ellipses and centroids included for group comparisons. These visualizations illustrated relationships between attributes and the three-dimensional space defined by PCA, helping to discern patterns of variability and mean shape differences among groups. Artifacts were categorized into predefined groups to explore the influence of blanks and techno-structural properties on tool morphology. These analyses utilized several tools from the assemblage variability panel in AGMT-3D, which is designed to test and describe variability within and between assemblages [20]. Shape variability is quantified as the mean multidimensional Euclidean distance of items in a group from the group’s centroid (mean shape). Centroid size is calculated as the square root of the sum of squared Euclidean distances from all landmarks to the artifact’s centroid [20].

To further explore variability, we employed the “group-mean distance calculator.” This tool computes the multidimensional Euclidean distances between group centroids, providing both a distance matrix and a dendrogram chart after selecting an attribute. This analysis facilitated comparisons between groups, helping to identify clusters of artifacts with shared morphological traits [20].

The “compare groups mean” tool facilitates the graphical comparison of mean shapes between two selected groups [20]. Finally, the “significance tester” allows for statistical evaluation of key differences between groups. This tool tests the equality of shape variabilities, the differences in mean shapes, and the differences in mean centroid sizes between two groups. The Wilcoxon rank-sum test, employed in AGMT3-D, is particularly suitable for analyzing 3D geometric morphometric data. This approach does not require normality or equal variances across groups, making it robust under conditions of high dimensionality and small sample sizes [134, 135]. Such statistical tests are increasingly applied in various research fields that encounter similar challenges, including economics, astronomy, and biomedical research [136, 137]. This methodological choice is consistent with best practices in the analysis of shape variability in archaeological and other contexts.

In our study, these tools were critical for quantifying intra- and inter-group variability and for assessing the influence of technological and typological attributes on tool morphology. For instance, the scatterplots generated by PCA not only illustrated group differences but also highlighted overlapping attributes, which were further explored using the group-mean distance calculator and other tools.

Through this comprehensive approach, we were able to identify patterns of variability that might otherwise go unnoticed. The integration of GPA, PCA, and the suite of tools within the assemblage variability panel provided a robust framework for analyzing shape differences, allowing for detailed comparisons across groups.

## Results

### Taphonomical data

**Fig 9:**
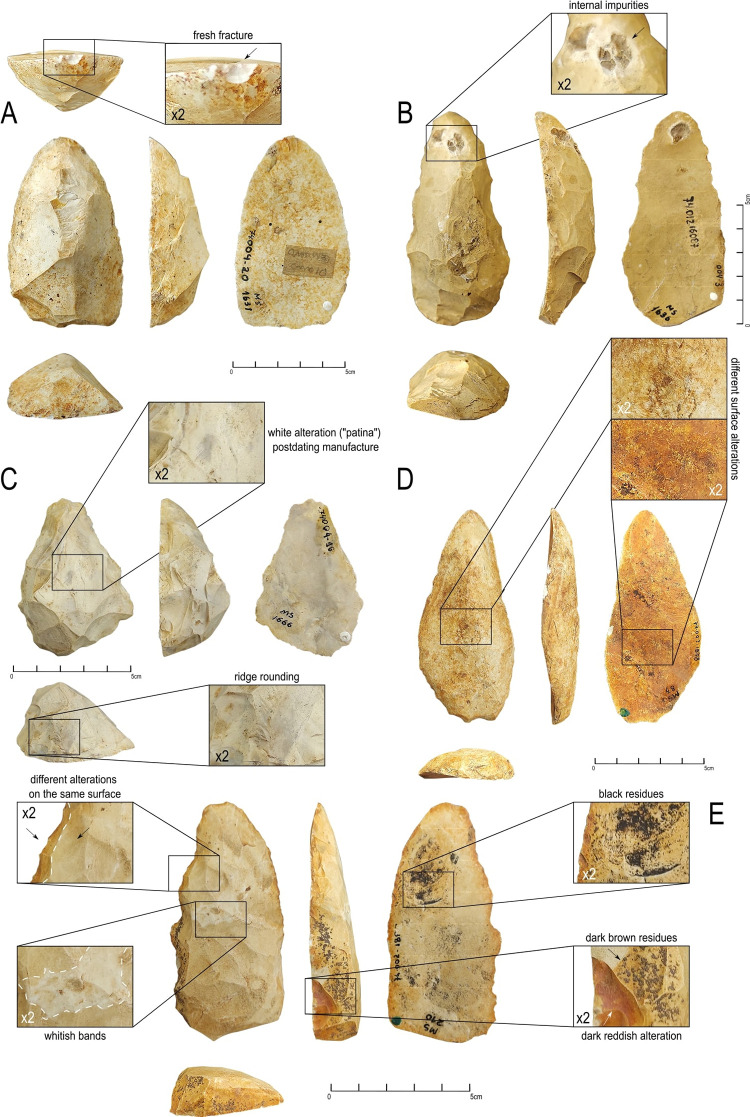
Some representative examples of surface conditions and taphonomic characteristics of GO-NI-01 collection. (A) fresh fracture, (B) internal impurities, (C) white alteration postdating manufacture, (D) different surface alterations and ridge rounding, (E) black residues and alteration.

A substantial majority of the artifacts, 56 out of 67 pieces (83.58%), are found to be complete. All artifacts are in a well-preserved and fresh condition, exhibiting sharp edges. However, in some instances, there are isolated rounding ridges (Fig 9C). The fragmented artifacts predominantly exhibit signs of post-depositional damage, as evidenced by the absence of significant counterblows and the presence of fresh patinas (Fig 9A). Significantly, all pieces manufactured from silexite (64 out of 67, accounting for 95.52%) display a characteristic white alteration or “patina” on their surfaces, which postdates their manufacture. This is typically indicative of a complex process involving phases of desilication and silica recrystallization, as outlined in Caux et al. [138].

Several of these silexite artifacts present a striking contrast, with one side featuring white patinas and the opposite side being more reddish or dark (Fig 9D). The dorsal side, being more exposed, is prone to chemical and biological alterations, leading to the development of a white patina. Conversely, the ventral side, which is relatively protected, largely preserves its original coloration. Therefore, the dorsal face of the flake blanks shows a lighter, off-white color, reflecting the natural hue of the silexite. This suggests that these objects have experienced minimal relocation since their knapping.

Additionally, other silexite artifacts exhibit peripheral marginal edges that are redder and darker compared to the creamier, whitish hue of the center/body of the piece (Fig 9E). The flat surfaces of these artifacts display an intermediate coloration. In some instances, macroscopic black residues are observed, particularly concentrated near the proximo-mesial parts of the artifacts (Fig 9E). This could indicate specific zones of these pieces that were buried over time while other parts remained exposed on the surface.

### Metrical characteristics

**Fig 10:**
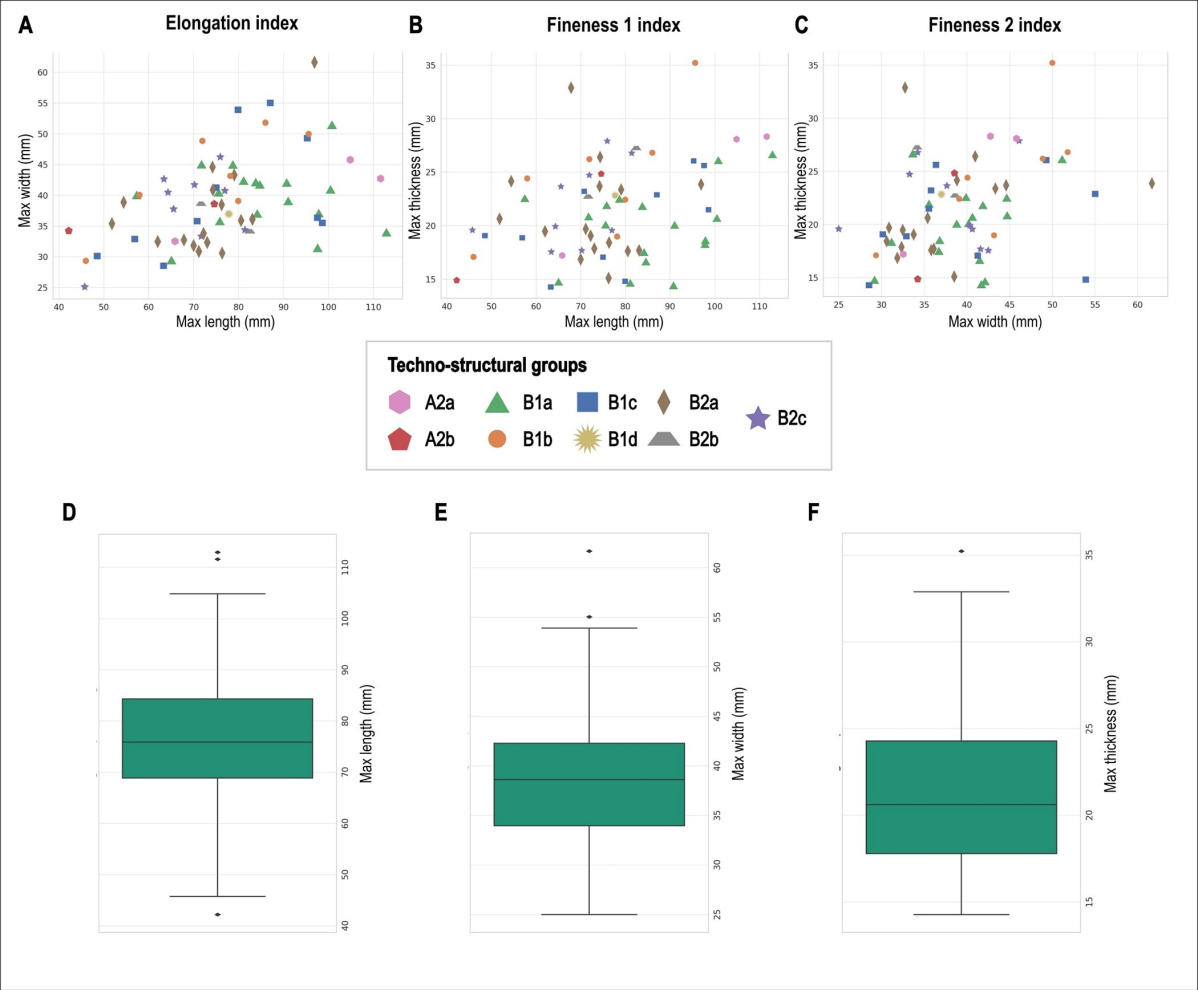
Dimensional distribution (length, width, and thickness) of the analyzed artifacts showed for techno-structural groups. (A) elongation index (B) fineness 1 index (C) fineness 2 index. Boxplot showing respectively length (D), width (E), thickness (F) of the techno-structural groups classes.

The lengths of the tools vary considerably, ranging from 42.22 mm to 112.9 mm, with an average length of approximately 76.82 mm (Fig 10D). The standard deviation of about 15.52 mm further points to a high variability in tool lengths. The width of the tools shows a more constrained range, from 25.01 mm to 61.65 mm, and an average width of around 39.05 mm (Fig 10E). The standard deviation of 6.97 mm for width is smaller than that for length, indicating less variability in tool widths. Thickness values range from 14.28 mm to 35.21 mm, with an average thickness of 21.34 mm (Fig 10F). The standard deviation of 4.49 mm for thickness, while less than that of length, is still significant, indicating a fair degree of variability. Additionally, the regression analysis provides crucial information about the presence or absence of allometry in the studied sample. The significant relationship between length and width (p < 0.001) indicates a trend in the joint variation of these two measures. This suggests the presence of positive allometry, meaning that as the artifacts increase in length, they also tend to increase in width. However, given the relatively low R^2^ value (0.195), this relationship is not very strong. On the other hand, the significant relationship between length and thickness (p = 0.010) also indicates the presence of allometry, although this relationship is weaker (R^2^ = 0.103). This suggests that longer artifacts tend to be slightly thicker, but this trend is not very pronounced. Finally, the significant relationship between width and thickness (p = 0.005) indicates that there is also allometry between these two measures. Wider artifacts tend to be thicker, but again, the strength of this relationship (R^2^ = 0.113) is moderate. The R^2^ values suggest that a considerable proportion of the variability in the measurements is not explained by allometry. This could be due to other factors such as structural or morpho-technological variability, which should be further explored in the future using 3D geometric morphometrics.

In examining the relationship between length and width across various tool groups, a diverse range of size ratios emerges (Fig 10A). Tool Group A2b presents the smallest tools in terms of both length (58.4 mm) and width (36.4 mm). Slightly larger in length but similar in width, Tool Group B2c (68.4 mm length, 37.9 mm width) and B2a (72.7 mm length, 37.5 mm width) demonstrate moderate size tools with a balanced length-to-width ratio. Tool Group B1b (73.7 mm length, 43.2 mm width) marks a departure with a notably wider profile compared to its length. As we move to Tool Group B2b (77.0 mm length, 36.4 mm width) and B1c (77.3 mm length, 39.9 mm width), there is a slight increase in length with varied widths. B1d (77.8 mm length, 37.0 mm width) presents a similar length to B1c but with a narrower width, aligning closer to the B2a and B2b groups in terms of width. Tool Group B1a stands out with a significant increase in length (85.2 mm) while maintaining a moderate width (39.4 mm), suggesting tools that are elongated but not excessively wide. Finally, Tool Group A2a showcases the longest tools in the dataset (94.1 mm in length) with a substantial width of 40.4 mm.

In exploring the length-to-thickness ratios (fineness 1 index) across the nine tool groups, we uncover a interesting spectrum of tool proportions (Fig 10B). Starting with Tool Group A2b, which has the lowest ratio at 2.94, these tools are characterized by their relative thickness in comparison to their length. Tool Group B1b follows closely with a ratio of 3.01, indicating a similar emphasis on thickness. Progressing to Tool Group B2b, with a ratio of 3.08, and B2c at 3.13, there’s a gradual shift towards tools that are slightly more elongated. This trend becomes more pronounced in Tool Group B1d, where the ratio jumps to 3.41, indicating tools that are longer relative to their thickness. B2a further accentuates this trend with a ratio of 3.45. Tool Groups B1c and A2a, with ratios of 3.80 and 3.84 respectively, mark a significant move towards tools that are much longer compared to their thickness. Culminating with Tool Group B1a, which has the highest length-to-thickness ratio of 4.32, this group clearly stands out for its elongated tools.

Exploring the width-to-thickness ratios across different tool groups reveals varied characteristics (Fig 10C). Starting with Tool Group B2b, which has the lowest ratio at 1.46, these tools are relatively thicker in comparison to their width. Moving to Tool Group B1d with a ratio of 1.62, and A2a at 1.65, we see a slight increase in the width relative to thickness, indicating tools that start to emphasize width a bit more. This trend continues with B2c at 1.73 and B1b at 1.77, where the tools are getting progressively wider in relation to their thickness. Tool Group B2a has a ratio of 1.78, further showing a preference for wider tools. Tool Group A2b, with a ratio of 1.83, marks a notable point where tools become significantly wider in comparison to their thickness. Tool Group B1c, with a ratio of 1.96, and B1a at nearly 2.00, tops the list, showing the highest emphasis on width relative to thickness.

From a metrical perspective, the differences among the nine techno-structural groups are particularly pronounced in the Fineness 1 index. However, Kruskal-Wallis tests for equal medians indicate that there are no significant differences in the medians of length, width, and thickness across the various techno-structural groups (Table 2; Fig 10). Furthermore, when conducting Mann-Whitney U tests comparing each techno-structural group’s dimensional parameters with those of other groups, we observe no statistically significant differences, with the exception of significant variations in lengths when comparing B1a to B2a and B2c (Table 3), and in thickness when B1a is compared to B2b. However, to mitigate the risk of inflating Type I errors and detecting spurious patterns, we applied p-value corrections to both the Kruskal-Wallis tests and the Mann-Whitney U test results. All corrected p-values resulted in 1, with few exceptions. This, combined with the fact that some of the techno-structural groups have low representativity, leads us to conclude that there are no statistically significant differences from a metric perspective in the studied sample.

**Table 2:** Data on techno-structural tools’ metrical dimensions.

	Length				Width				Thickness			
TSG	Mean Length	Min Length	Max Length	Std Length	Mean Width	Min Width	Max Width	Std Width	Mean Thickness	Min Thickness	Max Thickness	Std Thickness
A2a	94.09	65.84	111.59	24.69	40.36	32.54	45.80	6.94	24.53	17.21	28.31	6.34
A2b	58.40	42.22	74.57	22.87	36.39	34.24	38.54	3.04	19.84	14.86	24.82	7.04
B1a	85.25	57.48	112.90	14.19	39.43	29.18	51.14	5.32	19.74	14.28	26.52	3.64
B1b	73.65	45.99	95.60	16.87	43.19	29.36	51.79	7.86	24.45	17.09	35.21	5.95
B1c	77.28	48.51	98.59	17.57	39.88	28.55	55.02	9.64	20.34	14.30	26.05	4.19
B1d	77.83	77.83	77.83	0.00	37.00	37.00	37.00	0.00	22.85	22.85	22.85	0.00
B2a	72.68	51.82	96.91	10.78	37.50	30.60	61.65	7.76	21.05	15.10	32.88	4.47
B2b	77.03	71.64	82.41	7.62	36.40	34.17	38.63	3.15	25.00	22.73	27.26	3.20
B2c	68.38	45.74	81.38	10.47	37.92	25.01	46.09	6.28	21.86	17.49	27.83	3.91
Kruskal-Wallis test	p-value: 0.3286				p-value: 0.8467				p-value: 0.7166			

**Table 3:** Mann-Whitney U test results, conducted on principal measurements of unifacially shaped artifacts depending on their techno-structural groups. The table shows p-values for comparisons of variables (Length, Width, Thickness) between groups. Full confidence intervals for each comparison are available in S3 Table.

Variables	Techno-structural group																							
	Length								Width								Thickness							
	A2b	B1a	B1b	B1c	B1d	B2a	B2b	B2c	A2b	B1a	B1b	B1c	B1d	B2a	B2b	B2c	A2b	B1a	B1b	B1c	B1d	B2a	B2b	B2c
**A2a**	0.4000	0.4160	0.2670	0.2170	1.0000	0.2540	0.8000	0.1450	0.8000	0.5460	0.6670	0.9370	1.0000	0.3590	0.8000	0.6000	0.4000	0.2160	0.6670	0.2170	1.0000	0.4210	0.8000	0.4820
**A2b**		0.0700	0.3330	0.2730	0.6670	0.4710	0.6670	0.5820		0.3510	0.2220	0.9090	1.0000	0.8370	1.0000	0.6370		0.9470	0.5000	1.0000	1.0000	0.8370	0.6670	0.7270
**B1a**			0.1660	0.2190	0.6670	**0.0070**	0.3510	**0.0070**			0.2340	0.5980	0.7780	0.1170	0.3510	0.7460			0.0470	0.6700	0.3330	0.5520	**0.0470**	0.3320
**B1b**				0.7400	1.0000	0.6710	1.0000	0.2990				0.4750	0.5000	0.0890	0.2220	0.2100				0.1330	1.0000	0.1750	0.6670	0.6060
**B1c**					1.0000	0.4450	1.0000	0.3070					0.9090	0.6170	0.9090	1.0000					0.9090	0.7320	0.2730	0.3480
**B1d**						0.5880	1.0000	0.4000						0.8240	1.0000	0.8000						0.8240	1.0000	1.0000
**B2a**							0.6410	0.3800							0.8370	0.3800							0.2610	0.5150
**B2b**								0.3270								0.5820								0.4360

The average length in the collection is approximately 76.82 mm, and the average width is about 39.05 mm. This gives a length-to-width ratio of roughly 1.97, which is very close to the ideal ratio of 2:1 for lithic blades [86]. This close approximation suggests that many of the tools in the collection might fall under the category of blades in a morphological sense, adhering to this characteristic dimensional proportion. The wide range in lengths (42.22 mm to 112.9 mm) and widths (25.01 mm to 61.65 mm) indicates a diversity in the tool types. However, the average measurements suggest a strong presence of blade-like tools.

### Raw material and blanks used for the manufacture of unifacially shaped artifacts

The vast majority of unifacial pieces were produced on silexite (n = 64), with the remainder on silicified sandstone (n = 3). Various flake-supports were utilized, including flake 1A (n = 14), flake 1B (n = 10), flake 2 (n = 1), and flake ind (n = 42).

**Fig 11:**
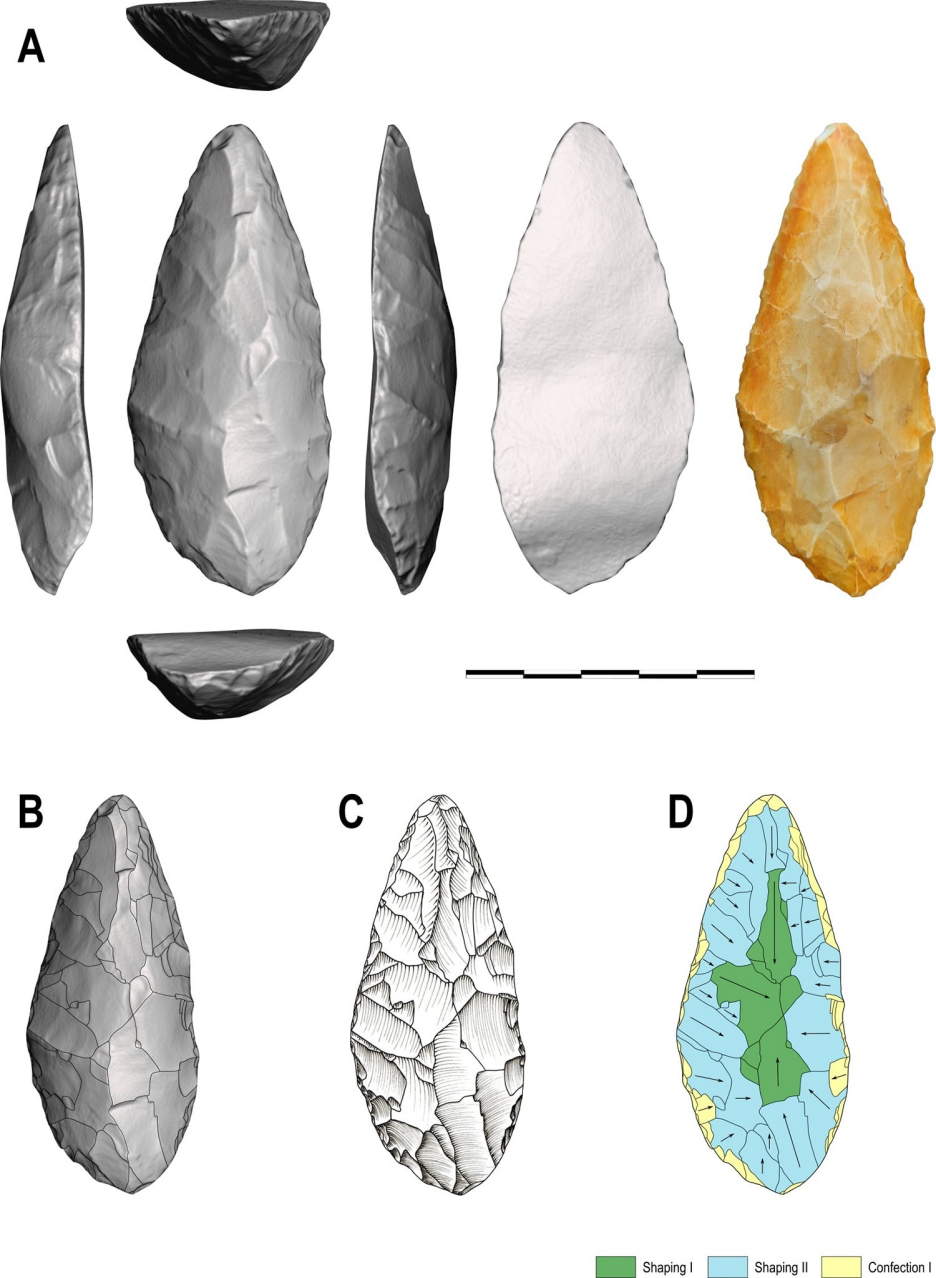
Unifacial tool on silexite flake with shaping operations covering the entire dorsal surface. Piece # MS84-74002-28 from Workshop 1, GO-NI-01: (A) 3D model; (B) shaping scars on 3D model; (C) manual technical drawing (by MGV) also assisted by the 3D model; (D) diacritic scheme and differentiation of shaping and confection phases.

It is noteworthy that the selection of certain flake blanks is associated with specific shaping modes. Notably, indeterminate flakes are preferred for shaping mode 2 (Fig 11). Flake 1A (n = 14) is also used for shaping mode 2 (n = 8), but shows a marked tendency towards shaping mode 4 (n = 6). Shaping modes 3 and 4 are also linked to flake-support 1B (n = 10). The flake-support 2, shaped by mode 1 (n = 1), constitutes an exception among these 67 unifacial pieces.

### Structural data

#### Techno-structural groups (TSG)

**Fig 12:**
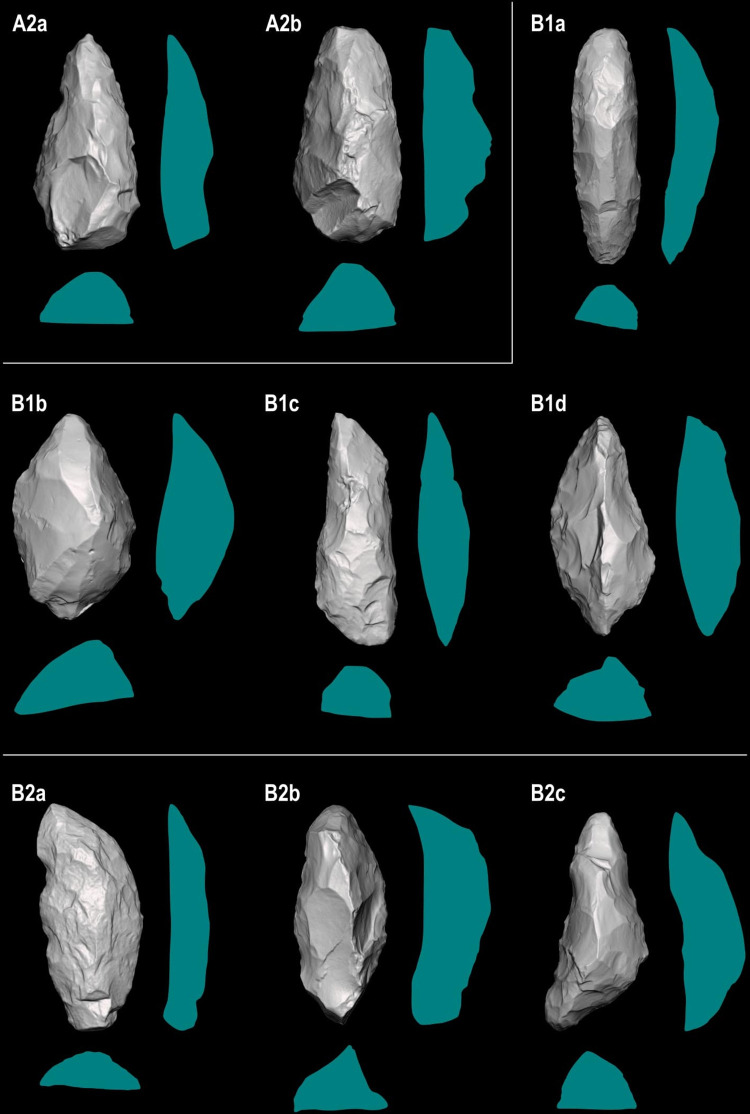
Techno-structural groups evidenced on samples’ 3D scans. In turquoise, the main transverse and longitudinal sections of each group are shown. Tool object with an asymmetric profile and a semi-circular or triangular cross-section (A2a and A2b); tool blank object with a symmetric profile, semi-circular, triangular, or trapezoidal cross-section (B1a, B1b, B1c, B1d); tool-blank object with an asymmetric profile, semi-circular, triangular, or trapezoidal cross-section (B2a, B2b, B2c).

**Fig 13:**
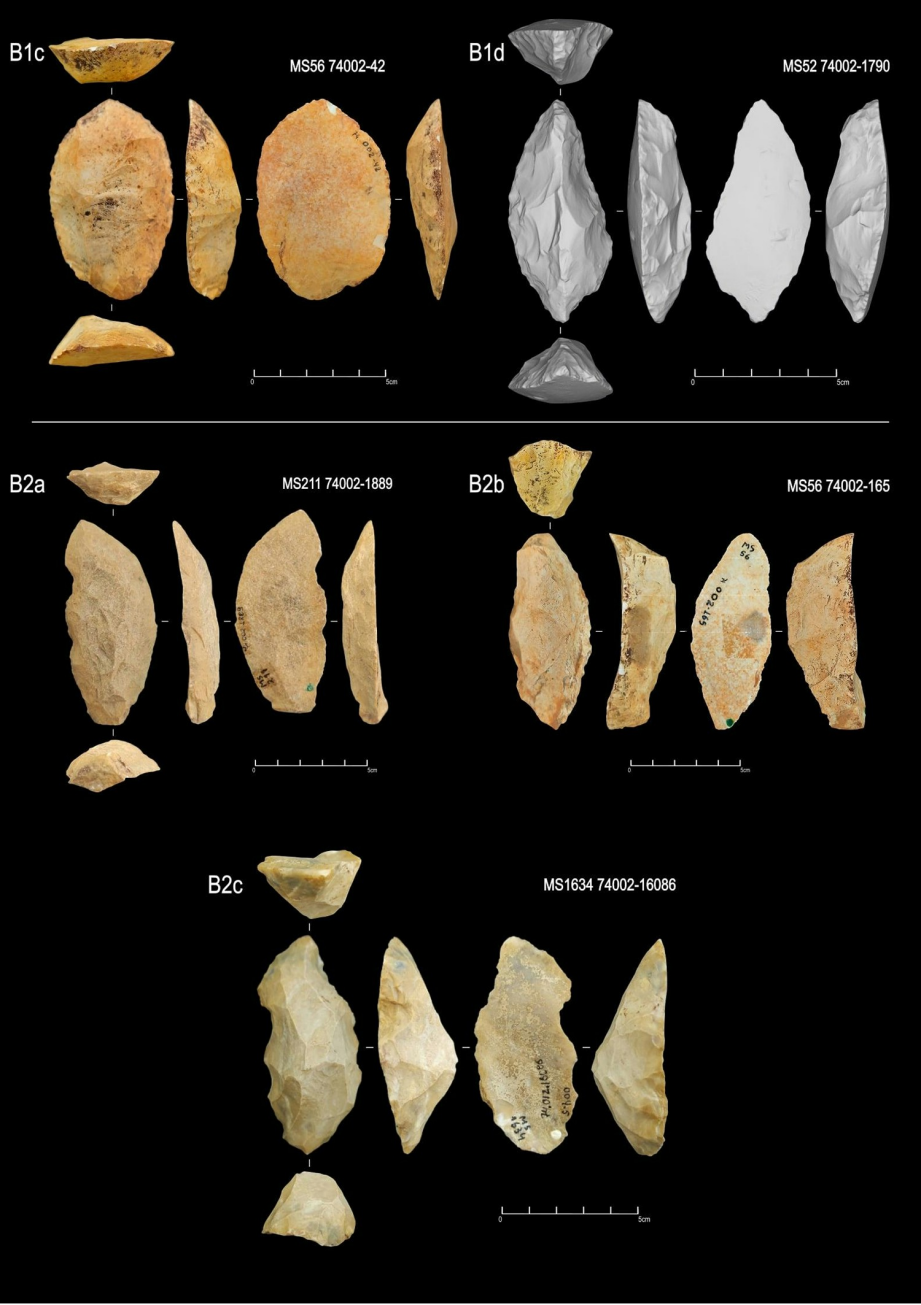
Examples of unifacial artifacts from GO-NI-01 site, Central Brazil.

**Fig 14:**
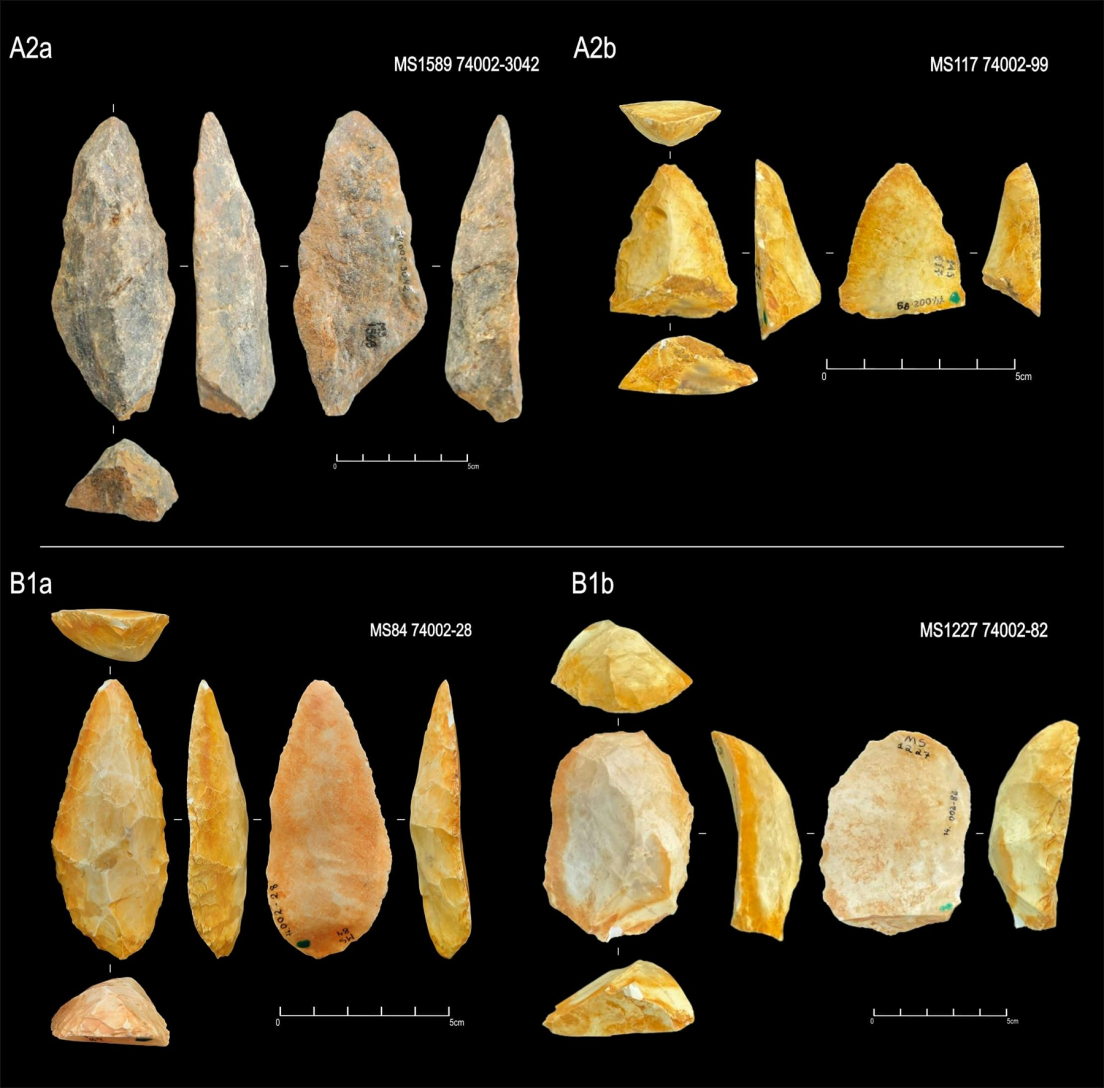
Examples of unifacial artifacts from GO-NI-01 site, Central Brazil.

Nine combinations of techno-structural features allowed to distinguish at least nine different techno-structural groups (TSG), which include some variants. As noted in the methodology flowchart (Fig 4) all TSG are characterized by the combination of a particular profile view and a particular cross-section (Fig 12). Examples of the variability internal to these groups are shown in Figs 13 and 14. It is crucial to emphasize here that we are discussing not techno-functional groups, but structural groups, meaning pieces that share a unique structure. This classification approach is chosen because using a broader array of qualitative criteria would have led to an overwhelming number of groups, complicating the identification of meaningful structural patterns. Therefore, we have opted for a reasoned classification, prioritizing structure over other morpho-technological criteria.

The techno-structural group A2a consists of three complete artifacts (Table 4), all belonging to GO-Ni-01 workshop 1. Two pieces are made of silexite and one of silicified sandstone. From the frontal view, triangular morphology predominates (n = 2) over pentagonal morphology. All these tools have an asymmetric profile, with two attributed to subtype 3 and one to subtype 1. They also share a semi-circular cross-section. Generally, the preferred flake blank (n = 2) remains undetermined, though one piece is associated with a flake type 2. A single artifact has a cortex of 25 to 50% located in its central part, along the morphological axis. Each piece presents different technological axes: proximal end struck, left side struck, and undetermined. This techno-structural group is predominantly (n = 2) shaped through Mode 2, the rest through Mode 1. All these pieces (n = 3) have a strictly flat surface. None of the pieces have a torsion line, inflection, or trace of reconfiguration. On the upper face of the artifact, the number of negatives is generally between 15 and 25 (n = 2), with only one below this range. The SDI (Scar Density Index) is 0.00 for two pieces and 0.02 for one. Two pieces have a striking platform. None of these three pieces are considered *limaces* according to the typology of Bordes [87], but as double convergent side-scraper (n = 1).

**Table 4:** Data counting the number of tools per tool group.

Tool group	n	%
A2a	3	4.47
A2b	2	2.98
B1a	17	25.37
B1b	7	10.44
B1c	10	14.92
B1d	1	1.49
B2a	16	23.88
B2b	2	2.98
B2c	9	13.43
**TOTAL**	**67**	**100.00**

The techno-structural group A2b includes two pieces attributed to GO-Ni-01 workshop 1, both fragmented and made of silexite. Each presents a different frontal view: triangular and teardrop. The profile is asymmetric in both cases, but the subtype differs from one piece to the other, one being associated with subtype 1 and the other with subtype 3. Both artifacts have a triangular cross-section. The flake blank of these pieces remains undetermined. One piece distinctly has a cortex, between 0 and 25% on the mesial part. The technological axis varies within the techno-structural group, with a left side struck axis (n = 1) and a left proximal oblique struck axis (n = 1). The two pieces are shaped according to mode 2. Their flat surface relief is strictly flat (n = 2). None of these pieces have a torsion or inflection line. One piece shows signs of reconfiguration. Negatives on the dorsal surface of both pieces are between 15 and 20 removals, and their SDI index is between 0.01 and 0.02. The striking platform is absent in the entire group. These pieces do not correspond to the definition of *limace* according to Bordes [87], though one piece is categorized as a double convergent side-scraper.

The techno-structural group B1a (n = 17) includes 17 complete and 2 fragmented pieces. The majority of these pieces (n = 16) are attached to the GO-Ni-01 workshop 1, the rest belonging to the GO-Ni-08 collection (n = 1). Almost all artifacts (n = 16) are made of silexite and one piece in silicified sandstone. Five types of frontal view morphologies are distinguished within this group: irregular (n = 3), triangular (n = 1), ovoid (n = 8), teardrop (n = 4), and ovoid-rectangular (n = 1). All pieces share a symmetric profile 1 and a semi-circular section. Although the type of flake blank could not be determined for most artifacts (n = 13), some pieces present a flake 1A type (n = 3), and only one piece a flake 1B type. None of the pieces show traces of cortex. The predominant technological axis is the proximal end struck (n = 6), followed by the distal end struck (n = 2). The vast majority of tools are shaped according to Mode 2, only one piece is associated with shaping mode 4. The reliefs of the flat surfaces can be strictly flat (n = 8), concave (n = 5), convex (n = 1), plano-convex (n = 1), and plano-concave (n = 2). The torsion line is present on three pieces, in oblique-distal (n = 1), oblique-mesial (n = 1), and oblique-proximal (n = 1) positions. Five pieces have an inflection line and five are reconfigured. All pieces have between 20 and 40 negatives of removals on the dorsal surface. One piece has an SDI of 0.00, eleven have an SDI of 0.01, and five of 0.02. Two tools out of the total artifacts have a remnant of striking platform. Only 3 pieces are considered *limaces* sensu Bordes [86], seven are double convergent side-scrapers and two backed side-scrapers.

The techno-structural group B1b brings together 7 pieces, six from the workshop 1 and 1 from workshop 3 of GO-Ni-01, the majority of which are complete (n = 6) and only one is fragmented. All artifacts are made of silexite. They all have an ovoid morphology in the frontal view. A majority of these artifacts (n = 6) have a symmetry 1 and only one has a symmetric profile 2. All pieces in this group have a triangular cross-section. A flake 1B type is predominantly used for these tools (n = 4). None of the pieces contain cortex. Three technological axes are distinguished in these pieces: proximal end struck (n = 4), right proximal oblique struck (n = 1), and left proximal oblique struck (n = 1). Three shaping modes are applied to the pieces: mode 2 (n = 3), mode 3 (n = 2), and mode 4 (n = 2). There are also 4 types of flat surface relief: strictly flat (n = 1), concave (n = 2), plano-concave (n = 2), and plano-convex (n = 1). Among the 7 pieces, several have an oblique-distal torsion line (n = 1), oblique-proximal (n = 1), and oblique-mesial (n = 1), the rest have no line. Two pieces have an inflection line. Only one piece shows signs of reconfiguration. All pieces have a number of removals scars on the dorsal surface between 10 and 30; presenting an SDI of 0.00 (n = 2) and 0.01 (n = 5). Among the 7 pieces, two have a striking platform. According to Bordes [86], three pieces in this group might be considered as *limaces*, two as backed side-scrapers, and one as a double convergent side-scraper.

The group of 10 complete pieces from B1c coming from GO-Ni-01 workshop 1 are all made of silexite. This group presents a wide diversity of frontal view morphologies: ovoid (n = 3), teardrop (n = 1), trapezoidal (n = 1), rhomboid (n = 1), triangular (n = 1), slug (n = 1), irregular (n = 1), and ovoid-rectangular (n = 1). Except for one piece with a symmetric profile 2, all share a symmetric profile 1. All pieces have a trapezoidal cross-section. Two types of flake blank are used in this group: flake 1A (n = 5) and flake 1B (n = 2). There are no traces of cortex on any of the pieces. The technological axes of the tools are very varied: left proximal oblique struck (n = 2), proximal end struck (n = 2), right proximal oblique struck (n = 2), distal end struck (n = 1), and left distal oblique struck (n = 1). Three types of shaping are used: mode 2 (n = 6), mode 3 (n = 2), and mode 4 (n = 2). Among the 10 pieces in this group, two have a torsion line, transverse-distal (n = 1) and oblique-mesial (n = 1); four have an inflection line and 4 artifacts are reconfigured. Negatives of removals on the dorsal surface range between 10 and 35. The SDI varies between 0.00 (n = 1), 0.01 (n = 7), and 0.02 (n = 1). The presence of a striking platform is noted on only one piece. Three pieces correspond to the term *limace* sensu Bordes [86], two to double convergent side-scrapers, one to backed side-scraper, and one to double side-scraper.

The techno-structural group B1d consists of a single complete piece of silexite from GO-Ni-01 workshop 1. It has a frontal teardrop morphology, a symmetry 1 profile, and an irregular cross-section on an indeterminate flake blank. It has no cortex and its technological axis remains undetermined. It was shaped using mode 2, and its lower surface has a convex relief. It shows no lines of torsion, inflection, or reconfiguration. With 23 removal scars on the dorsal surface, it has an SDI index of 0.01. It lacks a striking platform. Finally, it is considered a *limace* according to Bordes [86].

The techno-structural group B2a comprises a total of 16 artifacts, most of which are complete (n = 12), and 4 are fragmented. Except for two pieces from GO-Ni-01 workshop 3, all originate from workshop 1. One piece is made of silicified sandstone, the rest in silexite. From the frontal view, various morphologies are distinguished: triangular (n = 1), ovoid-rectangular (n = 3), teardrop (n = 3), pentagonal (n = 1), irregular (n = 2), and slug (n = 1). All pieces are associated with an asymmetric profile with sub-types 1 (n = 8), 2 (n = 2), and 3 (n = 6). All cross-sections in this group are semi-circular. Among the identified blanks, two pieces have a type 1A flake. There is no trace of cortex in this group. Various technological axes are identified, including distal end struck (n = 3), left side struck (n = 1), proximal end struck (n = 6), and undetermined axes (n = 6). There is a clear predominance of mode 2 shaping (n = 14), followed by mode 4 (n = 2). The reliefs of the flat surface are predominantly strictly flat (n = 9), although other reliefs are noted: flat-irregular (n = 1), convex (n = 3), and flat-concave (n = 3). Only two artifacts have a torsion line, oblique-distal (n = 1), and oblique-mesial (n = 1); four have an inflection line. Four tools show traces of reconfiguration. The number of removals scars on the dorsal surface ranges from 10 to 35. This group predominantly has an SDI of 0.01 (n = 12). Two pieces have a striking platform. Finally, no piece in this group can be categorized as a *limace* according to Bordes [86]; instead, there are convergent convex side-scrapers (n = 1), backed side-scrapers (n = 3), simple convex side-scrapers (n = 1), and double convergent side-scrapers (n = 2).

The pieces attributed to the techno-structural group B2b includes 2 complete silexite pieces from GO-Ni-01 workshop 1. From the frontal view, two pieces are ovoid-rectangular (n = 1) and ovoid (n = 1). Both have asymmetrical profiles of type 3 and a triangular cross-section. The flake blank used for both tools remain undetermined. There is no cortex on the pieces. Both artifacts are shaped using mode 2, and their technological axes are undetermined. Lower faces are flat-convex (n = 1) and concave (n = 1). Only one tool has a torsion line, all have an inflection line. Reconfiguration traces is noted in one piece. The range of scar removals on the dorsal surface is between 20 and 25. The SDI is 0.01 for both pieces in the group. None of the pieces have a striking platform. One artifact is considered a *limace* according to Bordes [86], the second is a double convergent side-scraper.

The last techno-structural group B2c has 9 silexite artifacts from workshop 1 (n = 8) and workshop 3 (n = 1) of GO-Ni-01. Seven of them are complete, two are fragmented. Four different frontal morphologies are distinguished: ovoid-rectangular (n = 2), slug (n = 2), ovoid (n = 3), and irregular (n = 2). All pieces have asymmetric profiles, but 3 sub-types emerge: sub-type 1 (n = 4), sub-type 2 (n = 4), and sub-type 3 (n = 1). The cross-section is always trapezoidal. Two types of flake blanks are used in this group: flake 1A (n = 3) and flake 1B (n = 2). No trace of cortex is found on the pieces of this group. The majority of the technological axes of the artifacts remain undetermined (n = 7), the rest have axes distal end struck (n = 1) and left distal oblique struck (n = 1). The shaping modes 2 (n = 3) and 4 (n = 6). The reliefs of the flat surface are predominantly strictly flat (n = 7). Only one piece has a torsion line in the oblique-proximal position and an inflection line. Three artifacts have been reconfigured. The number of scar removals on the dorsal surface ranges from 10 to 30. An SDI of 0.01 (n = 6) and 0.02 (n = 3) are identified. Only one tool retains a striking platform remnant. In this group, no piece is associated with the definition of a *limace* by Bordes [86]; identified are simple convex side-scrapers (n = 1), and backed side-scrapers (n = 1), with the rest of the pieces remaining undetermined.

### Techno-structural groups and morphological types

The artifacts identified as *limaces* by Bordes [87] consist of 11 tools out of a total of 67 artifacts, that is 16.42% (Table 5). These are categorized into five distinct techno-structural groups: B1a (n = 3), B1b (n = 3), B1c (n = 3), B1d (n = 1), and B2d (n = 1) (Table 5). All of these pieces are complete. They exhibit a wide variety of cross-sectional shapes: triangular (n = 4), trapezoidal (n = 3), semi-circular (n = 3), and irregular (n = 1). Similarly, the base flakes are quite diverse, including flakes 1A (n = 2) and flakes 1B (n = 2), with a tendency towards undetermined supports (n = 7). Interestingly, the majority of these limaces are shaped by mode 2 (n = 9), with the exceptions being modes 3 (n = 1) and 4 (n = 1). The preferred frontal view morphology is ovoid (n = 9), with the remaining being tear-drop shaped (n = 2). Most of these pieces have a symmetrical profile 1. Among these *limaces* as defined by Bordes, the predominant trend shows no striking platform (n = 10) neither a reconfiguration (n = 10), along with an SDI index of 0.01 (n = 10).

**Table 5:** Techno-structural groups (TSG) of tools showed for typological categories.

	Typology *sensu* Bordes (1981)															
	Limaces		Backed side-scraper		Convergent convex side-scraper		Double convergent side-scraper		Double side-scrapers		Simple convex side-scraper		Undetermined		TOTAL	
**TSG**	**n**	**%**	**n**	**%**	**n**	**%**	**n**	**%**	**N**	**%**	**n**	**%**	**n**	**%**	**n**	**%**
A2a	0	0.00	0	0.00	0	0.00	1	6.67	0	0.00	0	0.00	2	7.14	3	4.48
A2b	0	0.00	0	0.00	0	0.00	1	6.67	0	0.00	0	0.00	1	3.57	2	2.99
B1a	3	27.27	2	22.22	0	0.00	7	46.67	0	0.00	0	0.00	5	17.86	17	25.37
B1b	3	27.27	2	22.22	0	0.00	1	6.67	0	0.00	0	0.00	1	3.57	7	10.45
B1c	3	27.27	1	11.11	0	0.00	2	13.33	1	100.00	0	0.00	3	10.71	10	14.93
B1d	1	9.09	0	0.00	0	0.00	0	0.00	0	0.00	0	0.00	0	0.00	1	1.49
B2a	0	0.00	3	33.33	1	100.00	2	13.33	0	0.00	1	50.00	9	32.14	16	23.88
B2b	1	9.09	0	0.00	0	0.00	1	6.67	0	0.00	0	0.00	0	0.00	8	11.94
B2c	0	0.00	1	11.11	0	0.00	0	0.00	0	0.00	1	50.00	7	25.00	3	4.48
**TOTAL**	**11**	100.00	**9**	100.00	**1**	100.00	**15**	100.00	**1**	100.00	**2**	100.00	**28**	100.00	**67**	**100.00**

### 3D geometric morphometrics data

Geometric morphometric analyses were conducted using AGMT3-D software on 67 3D models. Overall, the morphological variability of TSG groups B2c, B2a, and B1c is greater than that of the other TSG groups (Table 6). This variability is quantified as the mean multidimensional Euclidean distance of the items in a group from the group’s centroid (i.e., the mean shape): the greater the value, the higher the variability [20]. TSG groups A2b, B2c, B2b, and B2a exhibit higher deviations from bilateral symmetry compared to other groups. In terms of deviation from bifacial symmetry, A2b, B1b, and B2b show the highest values. Regarding left edge curvature, the values are more or less uniform, with A2b having the highest value. Conversely, the B1b, B2c, and B2b groups exhibit the highest values in right edge curvature. In terms of left edge planform irregularity, A2b, B1c, and A2a present the highest values. Meanwhile, the B1d group has the highest value for right edge planform irregularity. As for section irregularity, the A2a group shows the highest value on the left edge, while the B2b group has the highest value on the right edge (Table 6).

**Table 6:** 3D statistical analysis on morphological variability for tool techno-structural groups (TSG).

TSG	N	Variability	Deviation from bilateral symmetry	Deviation from bifacial symmetry	Left edge curvature	Right edge curvature	Left edge planform irregularity	Right edge planform irregularity	Left edge section irregularity	Right edge section irregularity
**A2b**	2	7.18	**4.29**	**7.62**	**1.75**	0.75	**73.90**	35.27	30.59	52.75
**B1c**	10	**8.62**	3.77	5.17	1.04	0.98	**65.61**	41.02	61.99	52.53
**B1b**	7	7.24	3.56	**7.20**	**1.33**	**1.25**	52.39	40.53	70.25	46.27
**B2c**	9	**9.24**	**4.42**	6.91	1.06	**1.18**	58.78	43.14	48.81	66.22
**B2b**	2	5.28	**4.49**	**7.89**	0.69	**1.25**	38.67	40.69	68.19	**99.50**
**B1d**	1	0.00	2.91	6.46	**1.13**	0.91	12.47	**90.89**	29.60	79.08
**B2a**	16	**9.05**	**4.05**	4.94	0.75	1.09	44.11	42.99	77.34	59.04
**B1a**	17	7.20	3.96	5.27	0.77	0.84	45.50	41.79	47.80	57.86
**A2a**	3	4.86	3.98	5.55	0.56	0.62	**59.16**	41.24	**98.77**	46.07

**Fig 15:**
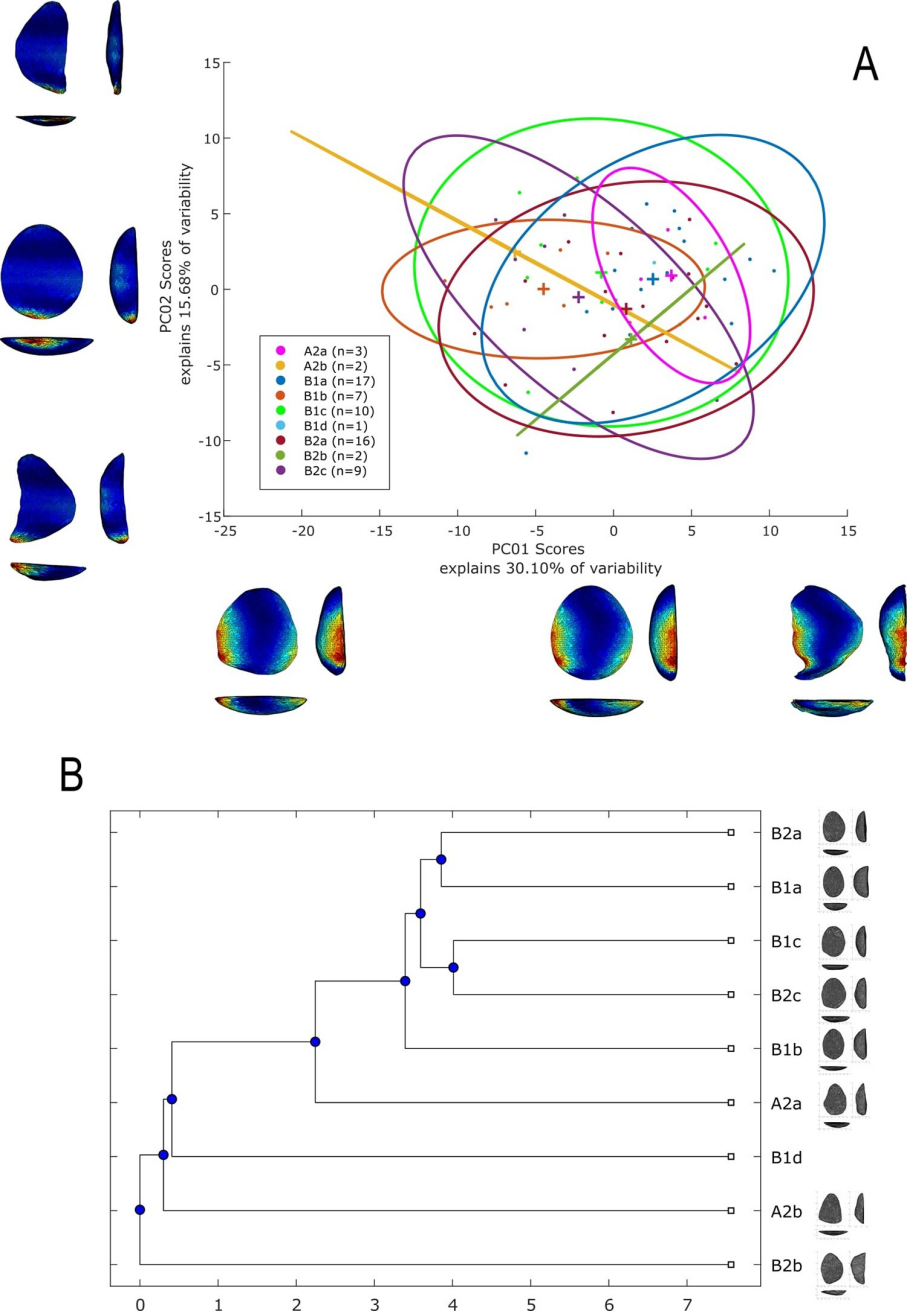
Statistical geometric morphometrics analysis on 3D models, compared for techno-structural groups (TSG). (A) scatter plot based on principal component analysis (PCA); the crosses indicate the localization of the TSG’ centroids within the PCA (B); main shape distance between TSG.

**Fig 16:**
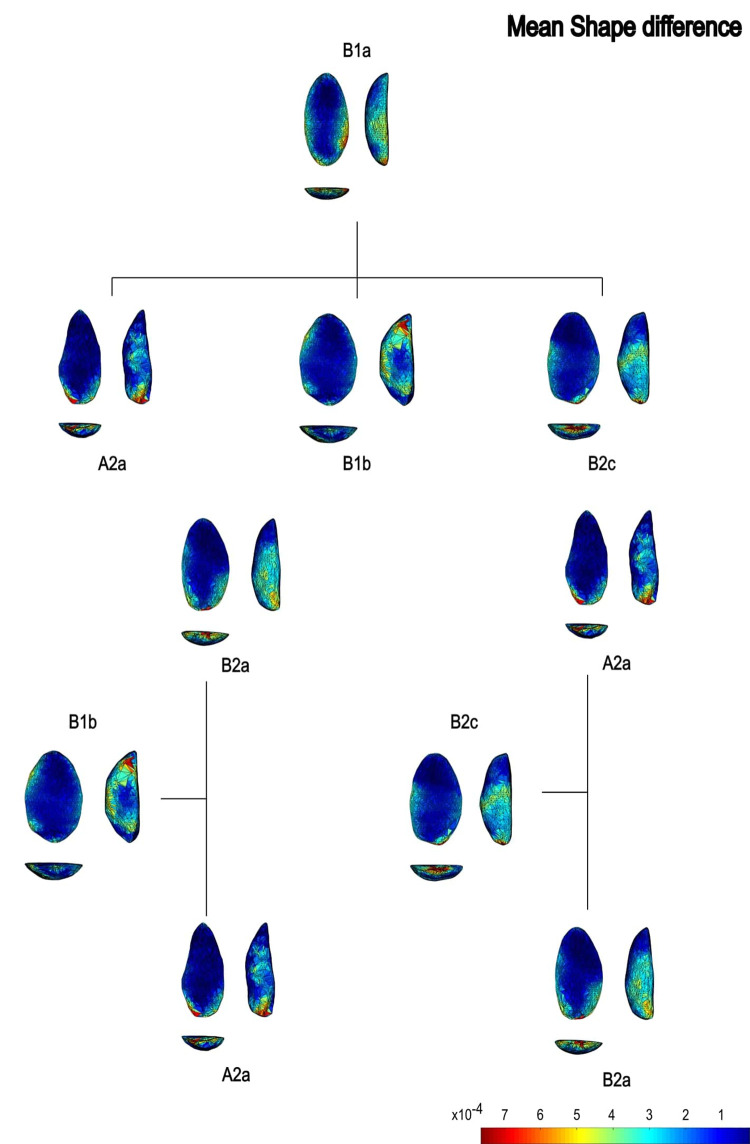
Comparison of significant mean shape differences between the techno-structural groups.

The PCA revealed morphological criteria with limited discrimination: the first two PCs only explained 45.79% of the overall shape variability, with the first 16 PCs expressing 90%. The main variation axis corresponded to a lateral edges deformation (PC 1) and a base deformation (PC 2), mostly from a frontal view with more or less convexity (PC1), and more or less pronounced inclination (PC 1 and 2), and to an expansion/thinning of the mesial profile (PC1) and base profile (PC 2). Fig 15 displays a significant diversity of three-dimensional morphologies associated with the TSGs, although there is also an overlap among the centroids of certain groups, such as B2a, B2c, and B1c, as well as between A2a and B1a. It is evident that A2a and B1b occupy a relatively restricted area of the shape space compared to the other groups. The A2a area corresponds to items with converging lateral edges and flat to convex ventral faces, which are more elongated. While B1b also falls within this shape space, the majority of pieces assigned to this group conform to more robust, thicker items with more convex lateral edges, yet maintaining a strictly flat lower surface. Similarly, the B2c group conforms to robust and thicker items with flat-concave lower surfaces. Furthermore, it is important to note that the results of Wilcoxon rank-sum tests on the Interpoint Distances between Group Means confirm that the mean shapes are significantly different between B1a and A2a, B1a and B1b, B1a and B2c, as well as between B1b and B2a, B1b and A2a, and between B2c and A2a, and B2c and B2a (Fig 16). This indicates a significant geometric diversity from a morphological perspective within the TSGs. However, we must exercise caution regarding group A2a due to its low representativeness.

**Fig 17:**
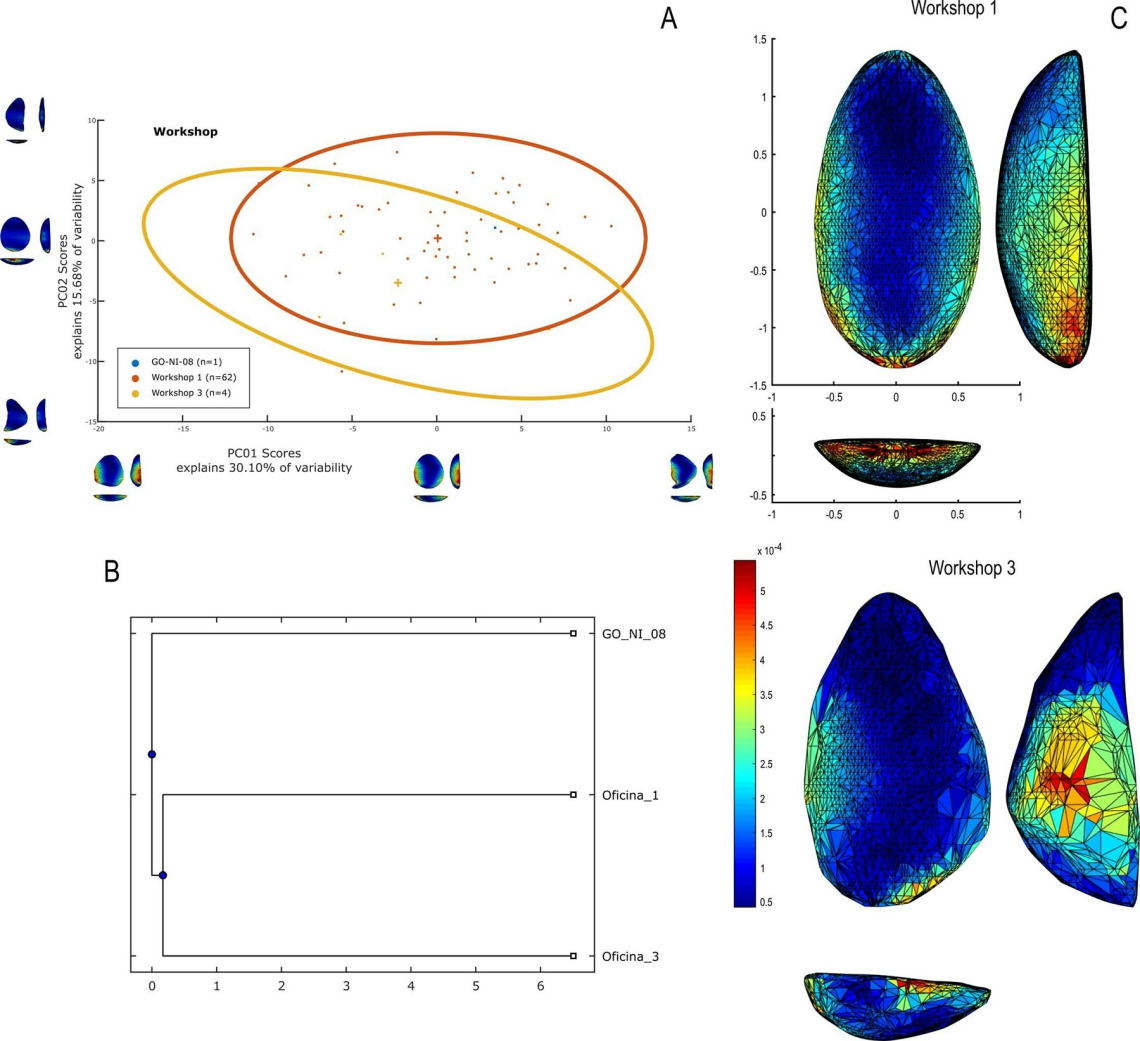
Scatter plot showing the variability of unifacially shaped tools groups for Workshop (A). Group distance for Workshop (B), and groups mean comparison between the mean shapes of Workshop 1 and Workshop 3 (C).

However, if we analyze the geometric morphological variability of the studied artifact population according to the workshop of origin (Fig 17), we observe that the pieces from Workshop 1 predominantly exhibit convergent convex distal edges in the frontal view, while pieces from Workshop 3 feature straight convergent edges. In the profile view, Workshop 1 piece’s exhibit Type 1 symmetry, whereas Workshop 3 pieces have an asymmetric profile with maximum thickness located at the base. Another notable characteristic is that the lower surfaces of the blanks from Workshop 1 are strictly flat, whereas those from Workshop 3 tend more towards a double concave-convex plane. In both workshops, however, there is a notable robustness in the volumetric structures. The results of the Wilcoxon Rank-Sum Test on Interpoint Distance between Group Means of Workshops 1 and 3 are significantly different, confirming these differences and the role of the pieces’ provenance in morpho-structural variability. This is despite the fact that the number of analyzed 3D models from Workshop 1 is substantially higher than the number of tools from Workshop 3. GO-Ni-08, having only one piece, is excluded from all considerations.

**Fig 18:**
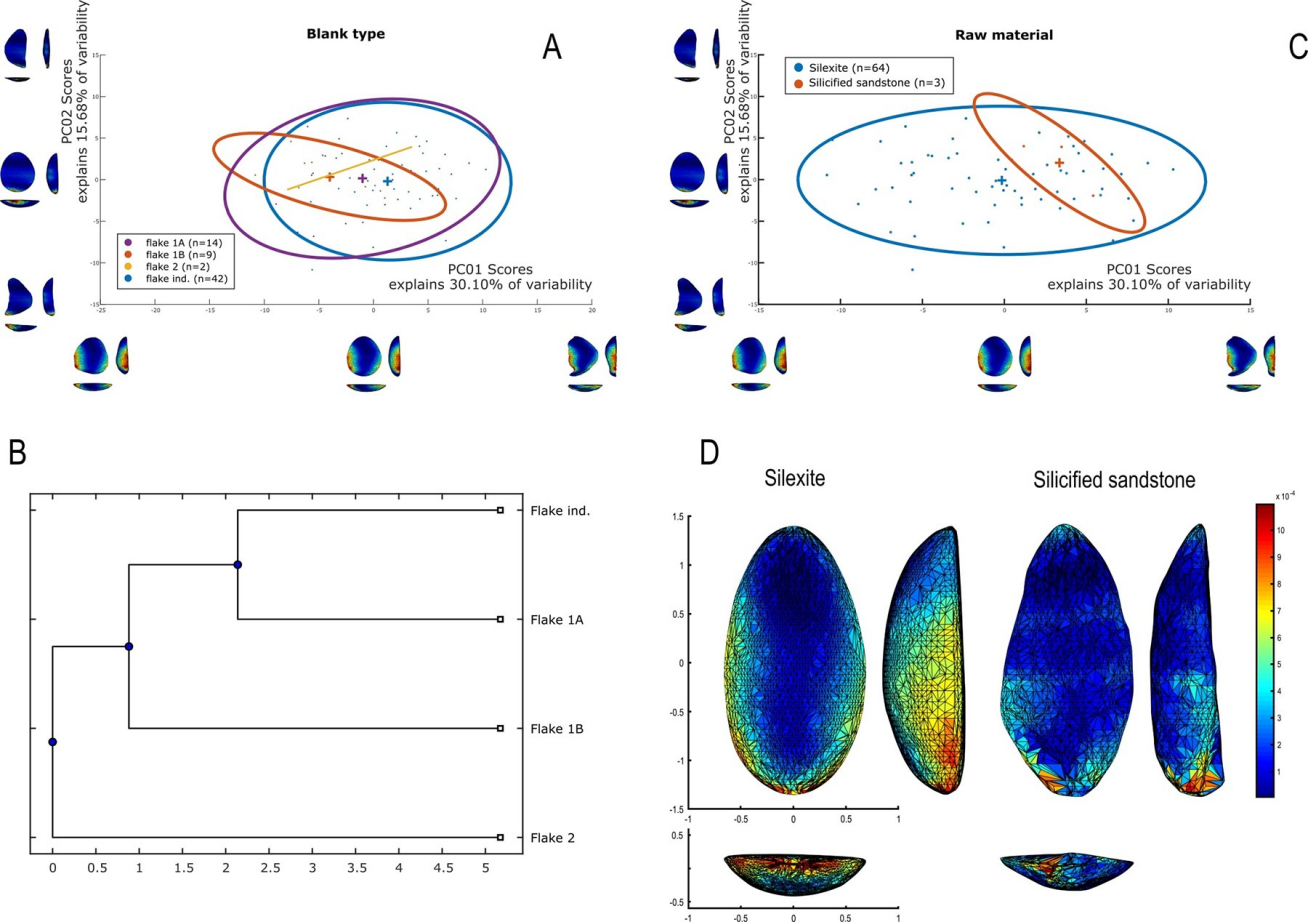
Scatter plot showing the variability of unifacially shaped tools groups for blank (A) and raw blank type (C), group distance for blank type (B), and groups mean comparison between the mean shapes of silexite and silificied sandstone (D).

In terms of the relationship between blank type and geometric morphology (Fig 18A), we observe a small differentiation between flake types 1B and 1A, despite a significant sharing of morpho-structural characteristics. Flake type 1B presents a slightly more robust structure than flake type 1A. Additionally, the former exhibits less convergent edges and less flat lower surfaces compared to the latter. The results of the Wilcoxon Rank-Sum Test on Interpoint Distance between Group Means of flake types 1B and 1A are significant, confirming these differences, and the role of the initial structure of the blank type in the morpho-structural variability of the studied population. On the other hand, examining the role of raw material (Fig 18C) reveals differences in morpho-structural attributes, despite some variability in sample sizes between groups. Thus, on one hand, Silexite items exhibit a structure very close to the traditional “limace”, with frontal and profile symmetry, convergent convex edges, and a strictly flat lower surface of the flake blank. On the other hand, Silicified sandstone items show a clear convergence of straight edges and pentagonal morphology in frontal view, as well as an asymmetric profile in side view, and a less regular lower surface of the blank compared to Silexite items. Additionally, in Silicified sandstone items, the maximum thickness is observed at the base of the piece, while in Silexite items, it is located in the mesial part. The results of the Wilcoxon Rank-Sum Test on Interpoint Distance between Group Means of Silexite and Silicified sandstone are significantly different, confirming these differences, and the role of raw material provenance in morpho-structural variability, despite the imbalance in the analyzed sample.

**Fig 19:**
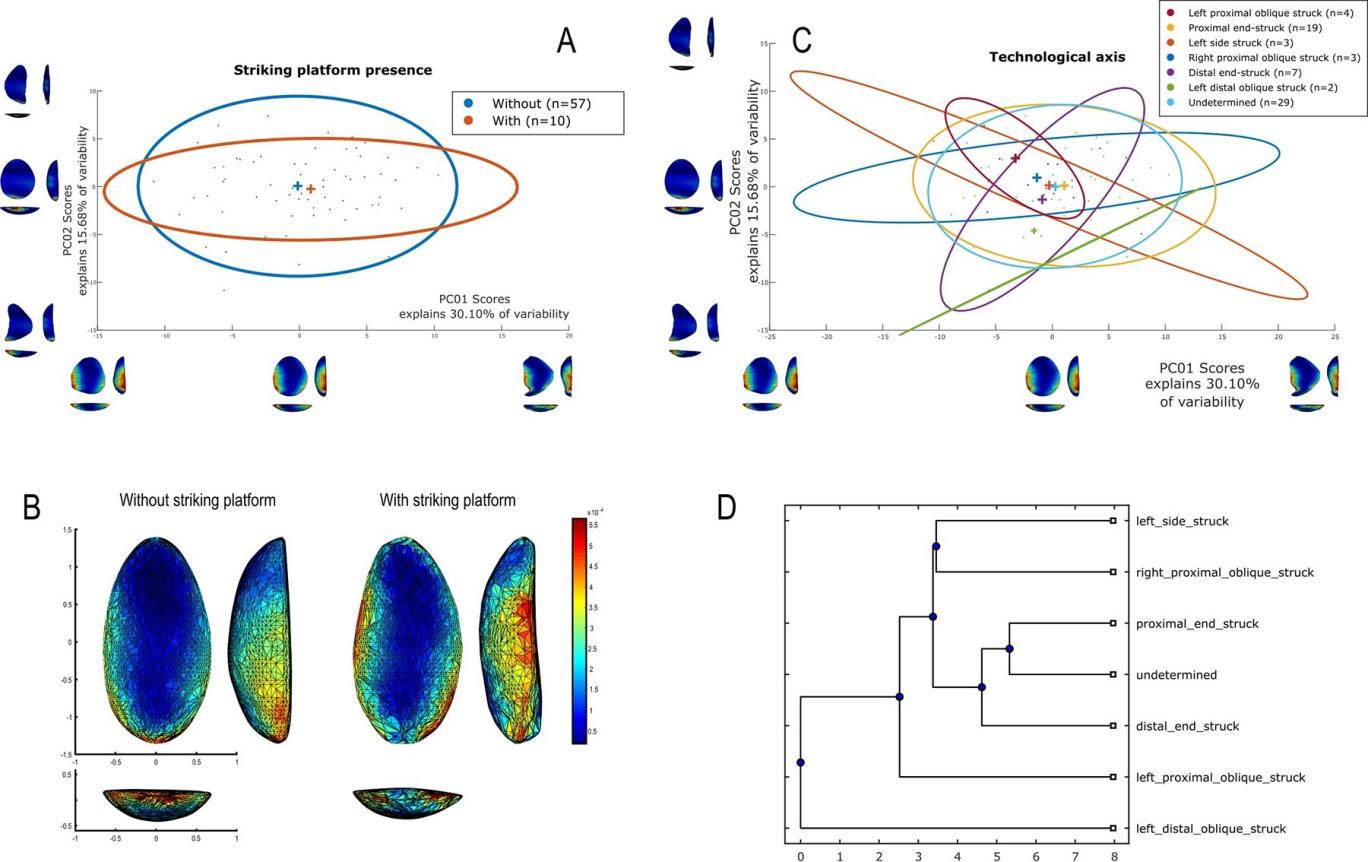
Scatter plot showing the variability of unifacially shaped tools for striking platform presence (A) and technological axis type (C), group distance for technological distance type (D), and groups mean comparison between the mean shapes of without and with striking platform presence (B).

Regarding the presence of striking platforms (Fig 19A), there is a clear difference in the volumetric structures of pieces with and without striking platforms. Pieces without striking platforms exhibit convergent convex edges, relative symmetry in frontal view, relative symmetry in profile view, and a strictly flat lower surface of the blank. On the other hand, pieces with striking platforms show asymmetry in both frontal and profile views. Additionally, the edge convergence in this group is less pronounced than in pieces without striking platforms. Also, the lower surface is flat-concave when a striking platform is present. However, both groups display more or less the same degree of robustness. Despite these observations, the results of the Wilcoxon Rank-Sum Test on Interpoint Distance between Group Means of pieces without and with striking platforms are not significantly different, which does not confirm these qualitative differences observed, suggesting instead a morpho-structural similarity in pieces with or without striking platforms. As for the technological axis (Fig 19C), most considered groups exhibit centroids very close to 0, except for the group of items with Left proximal oblique struck, which occupies a particular region in the three-dimensional morpho-geometric space. This group is characterized by asymmetry in frontal view, relative symmetry in profile view, and a rather convex lower surface of the blank. The results of the Wilcoxon Rank-Sum Test on Interpoint Distance between Group Means of the Left proximal oblique struck group and the other groups are significantly different, confirming these differences and the role of the technological axis in morpho-structural variability. This could indicate differences in methods of flake blank production, but caution is warranted given the sample studied.

**Fig 20:**
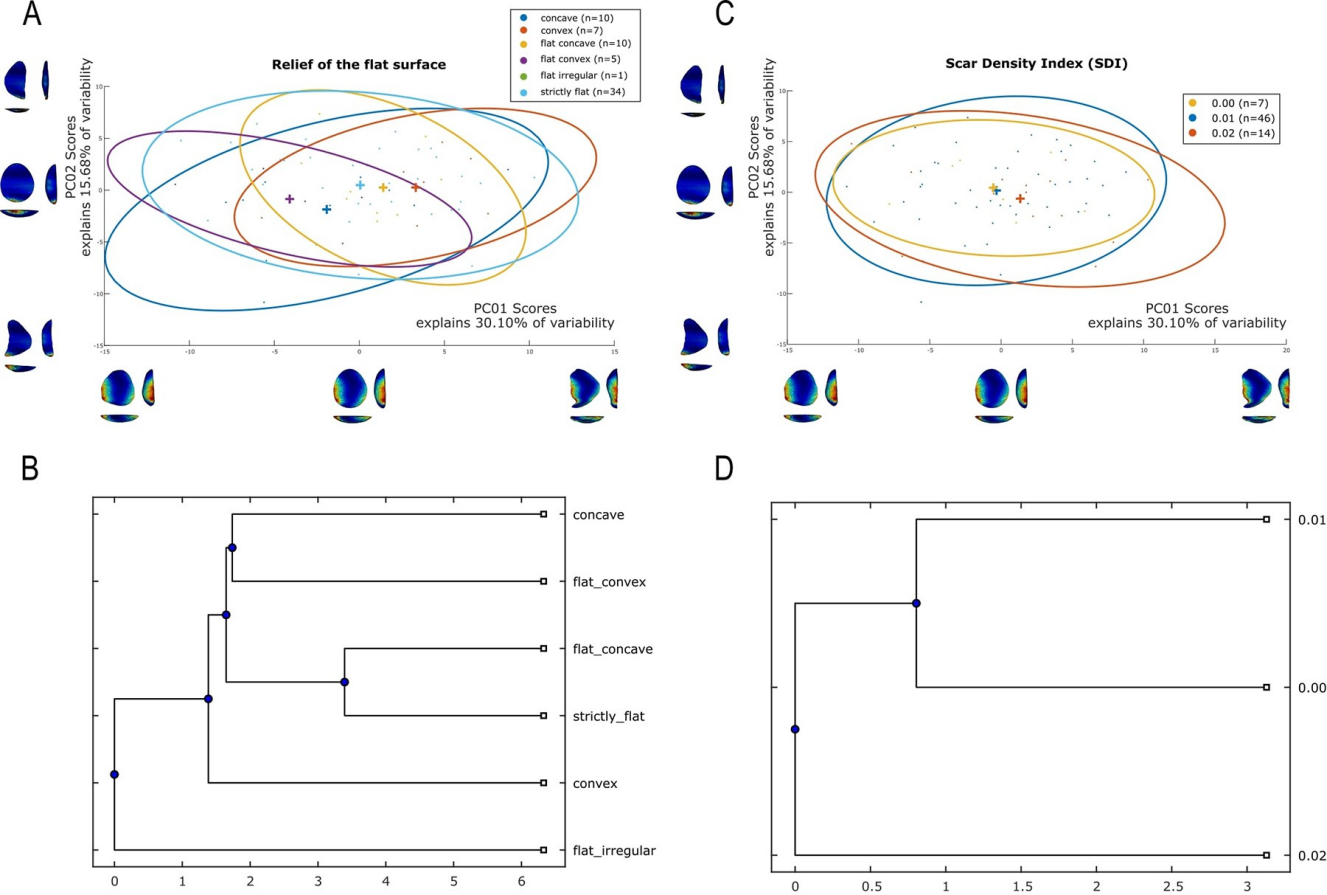
Scatter plot showing the variability of unifacially shaped tools for relief of the flat surface (A) and Scar Density Index (SDI) (C), group distance for relief of the flat surface type (B), and SDI (D).

In terms of the relief of the flat surface (Fig 20A), two groups of centroids are observed: on one hand, the flat concave and the concave groups, and on the other, the strictly flat, flat concave, and convex groups. In this respect, it appears that the flat convex items occupy a restricted space in the three-dimensional area. The group with a flat convex relief exhibits an asymmetric profile in both frontal and profile views, with a slight edge convergence in the distal part. Meanwhile, the group with a convex relief more closely resembles the classic limaces, with relative symmetry in frontal and profile views, and more significant convergence of distal edges. The results of the Wilcoxon Rank-Sum Test on Interpoint Distance between Group Means of flat convex and convex groups are significantly different, confirming these differences, and the possible role of a different conception of the lower surface of the flake blank in morpho-structural variability. This could, of course, indicate differences in flake blank production methods, as well as in the functional organization of production objectives. As for the SDI (Fig 20C), despite the centroids of the three groups being close to 0, the group with an SDI of 0.02 appears to differ minimally from the rest. Indeed, the group with an SDI of 0.02 presents clear, nearly straight convergent edges in the distal portion, as well as relative symmetry in profile view and a strictly flat lower surface of the blank. On the other hand, taking the group with an SDI of 0.00 as the comparison group, we observe that this group does not exhibit as significant a convergence as the previous group, in addition to asymmetry in frontal and profile views, and a flat-convex lower surface of the flake blank. Despite these qualitative observations, the results of the Wilcoxon Rank-Sum Test on Interpoint Distance between Group Means of the 0.02 SDI and 0.00 SDI groups are not significantly different, which does not confirm these observed qualitative differences, and suggests instead a morpho-structural similarity in pieces with different SDIs.

**Fig 21:**
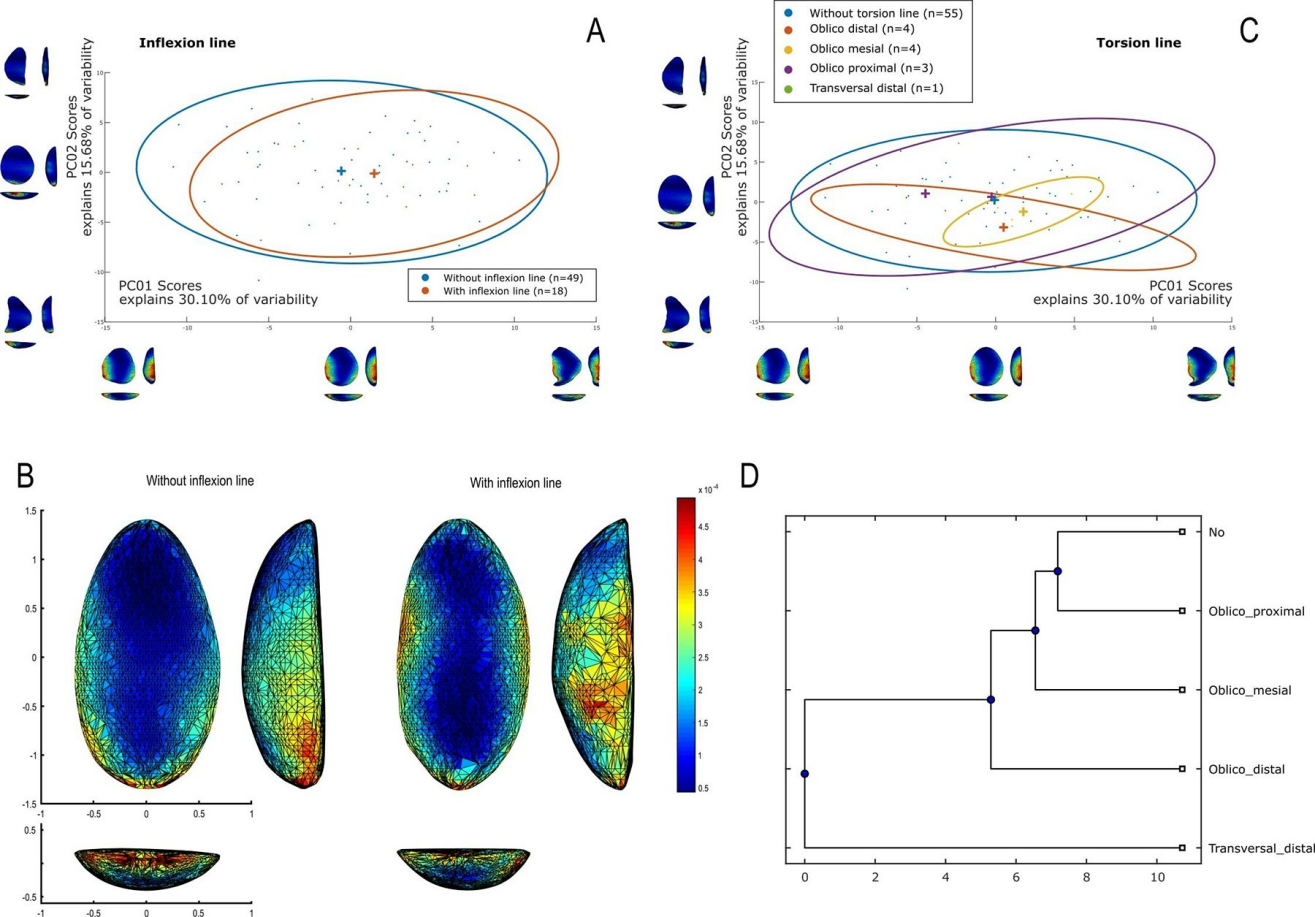
Scatter plot showing the variability of unifacially shaped tools for inflexion line (A) and torsion line presence (C), group distance for torsion line presence (D), and groups mean comparison between the mean shapes of without and with inflexion line (B).

Regarding the role of the inflection line (Fig 21A), we observe that the centroids of the two considered groups are very close to 0, suggesting a significant similarity. The results of the Wilcoxon Rank-Sum Test on Interpoint Distance between Group Means of Without and With inflection line groups are not significantly different, confirming this similarity, and suggesting a morpho-structural similarity in pieces with or without an inflection line. On the other hand, examining the role of the presence and location of a torsion line (Fig 21C), we find that the ’oblique proximal’ group occupies a reduced space in the three-dimensional area. Pieces in this group exhibit asymmetry in both frontal and profile views, as well as a convergence of convex edges in the distal section and a slightly flat-concave lower surface of the flake blank. In contrast, pieces without a torsion line resemble the classic double convergent side-scrapers of the morphological typology, with asymmetry in frontal view and slight symmetry in profile view, as well as a strictly flat lower surface of the flake blank. The center of mass differs from one group to another, suggesting potential different functions. Despite these qualitative observations, the results of the Wilcoxon Rank-Sum Test on Interpoint Distance between Group Means of ’Oblique-mesial’ and ’Without’ torsion line groups are not significantly different, which does not confirm these observed qualitative differences, and suggests a morpho-structural similarity between these groups. However, the same statistical test suggests a significant difference between pieces with a torsion line in oblique-mesial position and those with a torsion line in oblique-proximal position. In the latter, the position of the torsion line determines the location of the maximum thickness and the center of mass of the piece, suggesting potentially different functions. The same is true between the ’Oblique-mesial’ and ’Oblique-distal’ groups, which also show statistically significant differences. The structure of this last group tends to be more symmetrical in profile view and much more robust in terms of size.

**Fig 22:**
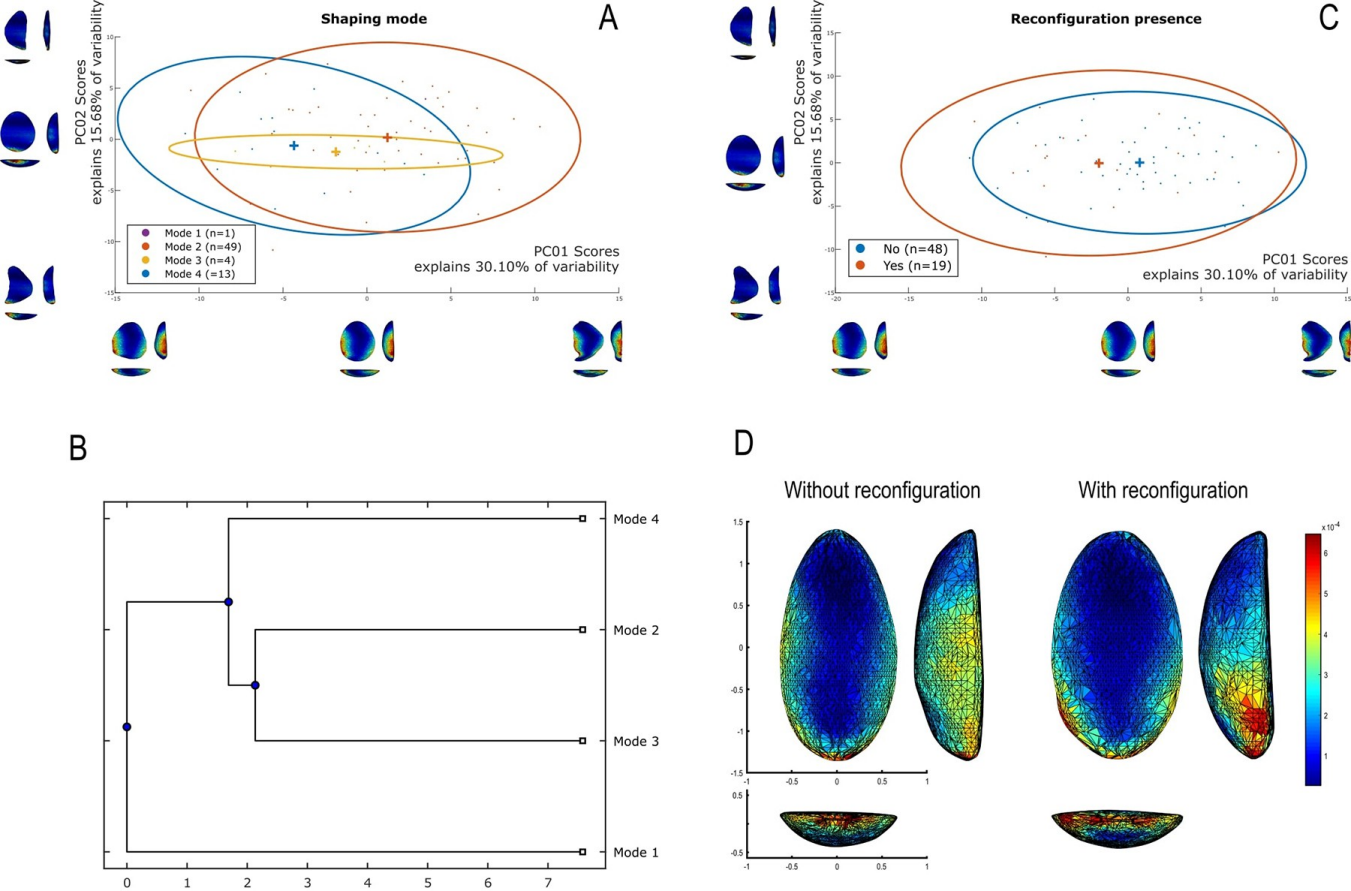
Scatter plot showing the variability of unifacially shaped tools for shaping mode (A) and reconfiguration presence (C), group distance for shaping mode (B), and groups mean comparison between the mean shapes of without and with reconfiguration (D).

Regarding the shaping mode (Fig 22A), we observe that pieces shaped using Mode 3 occupy a restricted space in the three-dimensional area. Pieces in this group exhibit asymmetry in both frontal and profile views, with the lower surface of the flake blank tending towards concave. Conversely, if we consider the Mode 2 group for comparison, we find that the pieces in this latter group are comparable to the double convergent side-scrapers of the Bordes typology, with asymmetry in frontal view and relative symmetry in profile view, and a strictly flat lower surface of the flake blank. The maximum thickness and the center of mass are differentially distributed between the two groups, in a mesio-distal position in the first, while in a mesio-basal position in the second. The results of the Wilcoxon Rank-Sum Test on Interpoint Distance between Group Means of Mode 3 and Mode 2 are significantly different, confirming these differences, and the important role of shaping modes in morpho-structural variability. Additionally, there is also a statistically significant difference between Mode 3 and Mode 4, with items from the former group being less robust than those belonging to the latter group. These results could suggest differences in the production methods of unifacially shaped tools, and potentially structured savoir-faire, but caution is warranted given the sample studied. As for the presence of reconfiguration, groups with pieces with and without reconfiguration traces present centroids very close to each other and to 0, suggesting indistinguishable structural patterns (Fig 22C, 22D). The results of the Wilcoxon Rank-Sum Test on Interpoint Distance between Group Means of groups Without and With reconfiguration traces are not significantly different, suggesting a morpho-structural similarity in pieces with or without reconfiguration schemes.

**Fig 23:**
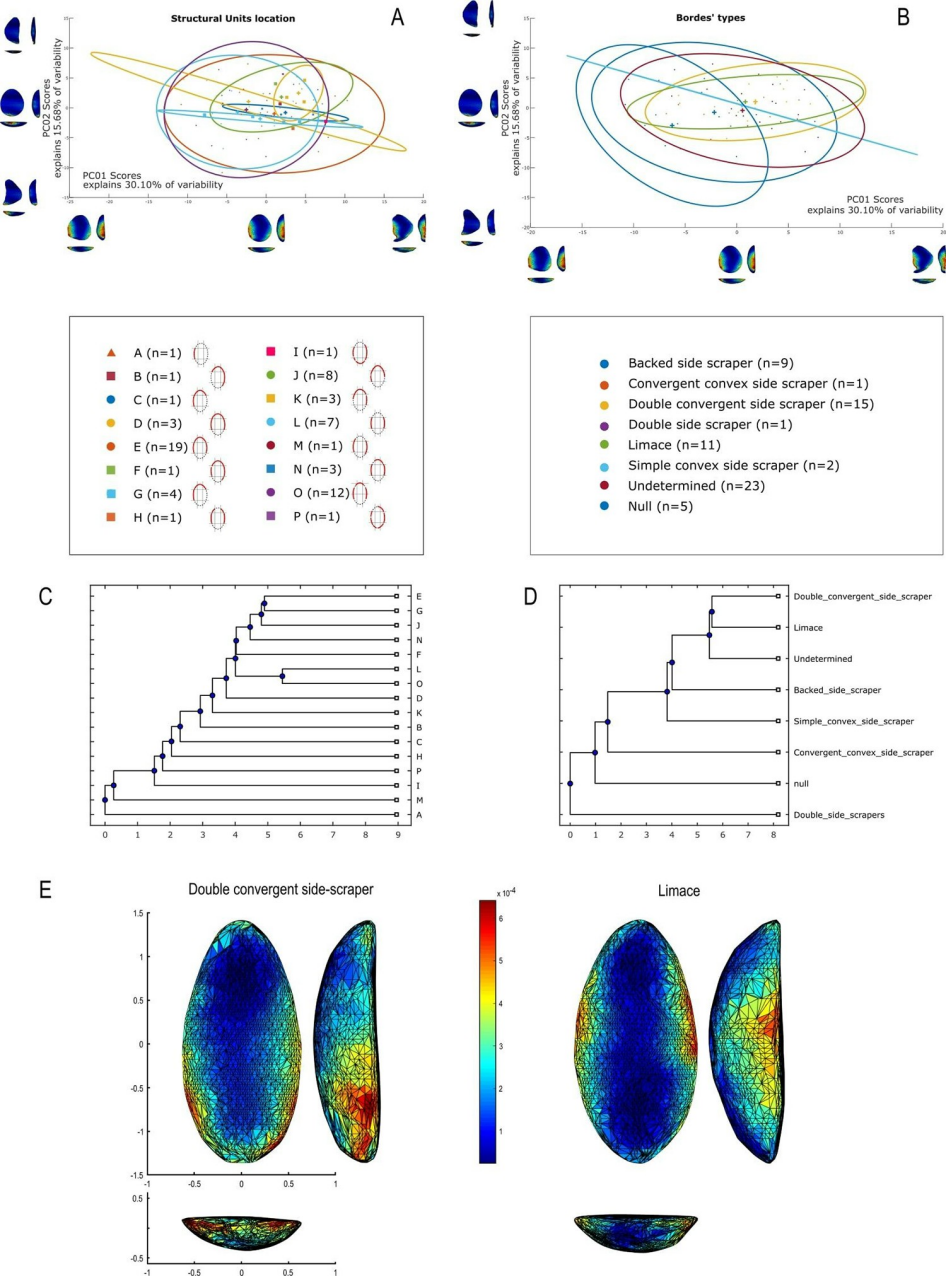
Scatter plot showing the variability of unifacially shaped tools for Structural Units (SU) location (A) and Bordes’ types (B), group distance for SU location (C), group distance for Bordes’ types (D), and groups mean comparison between the mean shapes of double convergent side-scraper and limaces sensu Bordes typology (B).

**Fig 24:**
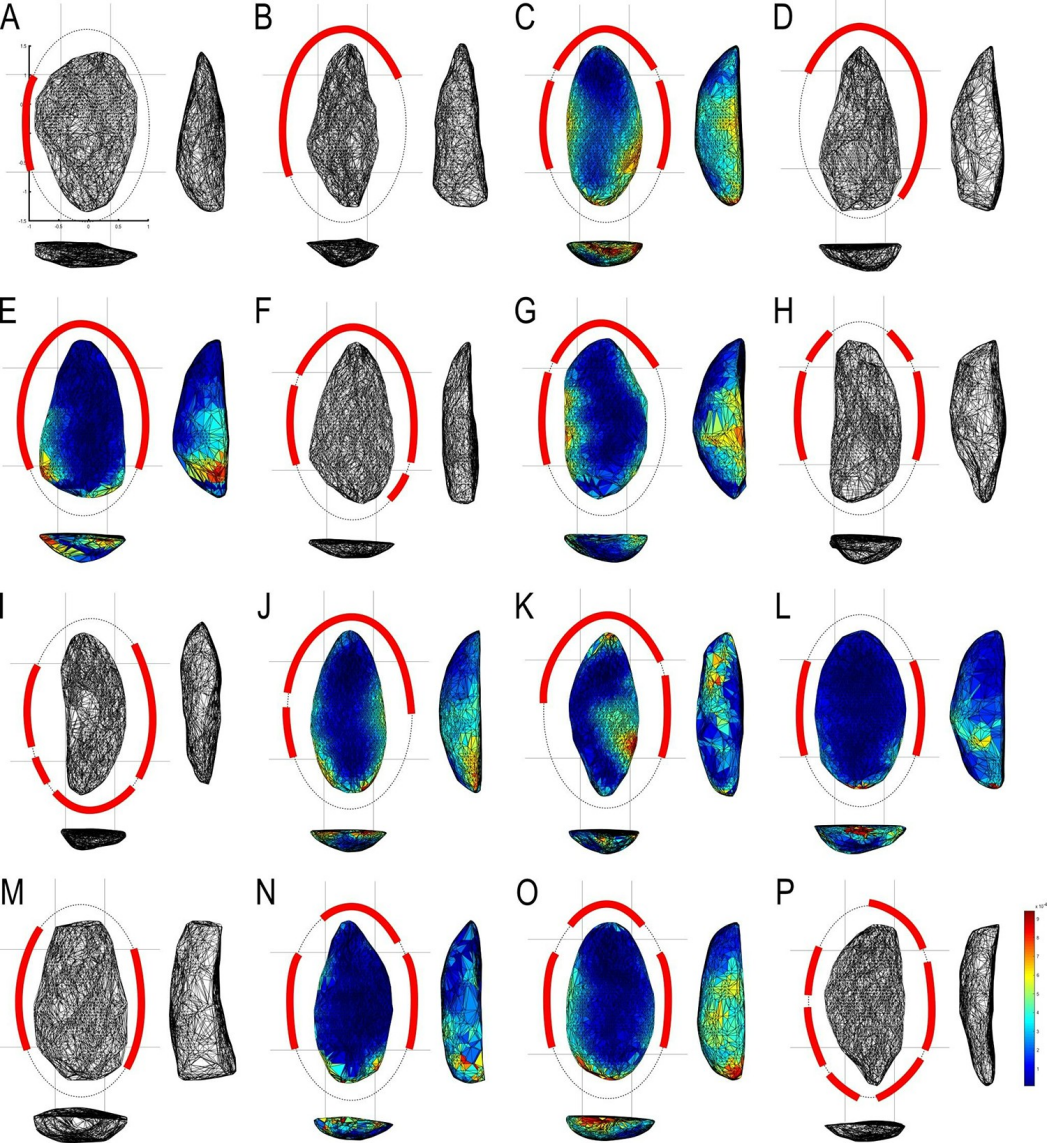
Variability of mean shapes and location of Structural Units (SUs). In red lines, the number indicating SU location. Groups A, B, D, F, H, I, M, and P each comprise a single piece. The remaining groups contain more than one piece, allowing the colors to reveal the location of internal variability in their shapes: the redder the area, the greater the variability.

In terms of Structural Units (SU) location (Fig 23A), at least two major groups of centroids can be observed, with sub-groups occupying restricted spaces in the three-dimensional area. Considering Group K (2 SUs) and Group O (3 SUs), it is evident that they differ significantly both in frontal and profile views, as well as in the relief of the lower surface of the flake blank. The results of the Wilcoxon Rank-Sum Test on Interpoint Distance between Group Means of these groups are significantly different, confirming the previously noted qualitative differences and suggesting an important role of the SU location in explaining the morpho-structural variability of the studied population. If we consider pieces that are tool objects and tool-blank objects, we note that tool objects exhibit common characteristics such as asymmetry in both frontal and profile views, convergence or non-convergence of edges in frontal view, convergence of edges in profile view, and maximum thickness in the proximo-mesial part, along with a variable lower surface of the flake blank. On the other hand, the quantitatively larger group of tool-blank objects generally presents relative symmetry in both frontal and profile views, convex or straight converging edges at both ends of the piece, and generally a maximum thickness in the mesial part, along with a generally flat lower surface of the flake blank. Fig 24 details this variability of mean shapes and SU location.

Finally, regarding the typology of Bordes [86], i.e., the role of the final product’s silhouette in the morpho-structural variability of the considered population, we can note that limaces and double convergent side-scrapers occupy similar spaces in the three-dimensional area, distinguishing themselves from other types (Fig 23B). Comparing limaces with backed side-scrapers, the most notable differences are in the asymmetry in profile view of the backed side-scrapers, as well as in the frontal asymmetry and differential distribution of maximum thickness. Despite these qualitative observations, the results of the Wilcoxon Rank-Sum Test on Interpoint Distance between Group Means of ’Limaces’ and ’Backed side-scrapers’ groups are not significantly different, which does not confirm these observed qualitative differences, suggesting instead a morpho-structural similarity between these typological groups.

## Discussion

This work presents a thorough research of Holocene unifacial tools from the GO-Ni sites in Central Brazil. The primary aim was to explore the determinants behind the variability within and between these tools. At the outset of our research, building upon insights previously highlighted in other studies, we hypothesized that this variability is a result of a combination of factors, including raw material availability and the functional and ergonomic requirements of the prehistoric inhabitants. Utilizing a pioneering method that integrates 3D geometric morphometrics with detailed techno-structural analysis, we sought to precisely quantify tool morphology and uncover their functional potential and adaptability. This approach facilitated an in-depth examination of the relationship between tool geometry, silhouette, volumetric attributes, and their technological and ergonomic implications.

Our findings reveal a high rate of artifact completeness and well-preserved condition for an open-air site. The occurrence of white patina on silexite artifacts, a sign of post-depositional processes like desilication and silica recrystallization, which could limit the scope of a manual analysis, did not do so in the case of 3D analysis. The coloration difference between the dorsal and ventral sides of artifacts suggests limited movement post-abandon. In this context, the observed variability in tool geometries across nine techno-structural groups (TSGs) indicates a range of probable functional needs and associated technological strategies. The close alignment of the average length-to-width ratio with the ideal ratio for blades suggests a dominance of blade-like tool technology in the sample. The 3D shape diversity within the TSGs, while always considering the low representativeness of some of them, challenges traditional morpho-typological classifications and highlights the presence of flexible shaping technology probably adapting to functional needs in Central Brazil during the Holocene.

### A new 3D techno-structural typology of unifacially shaped tools versus traditional typologies

**Fig 25:**
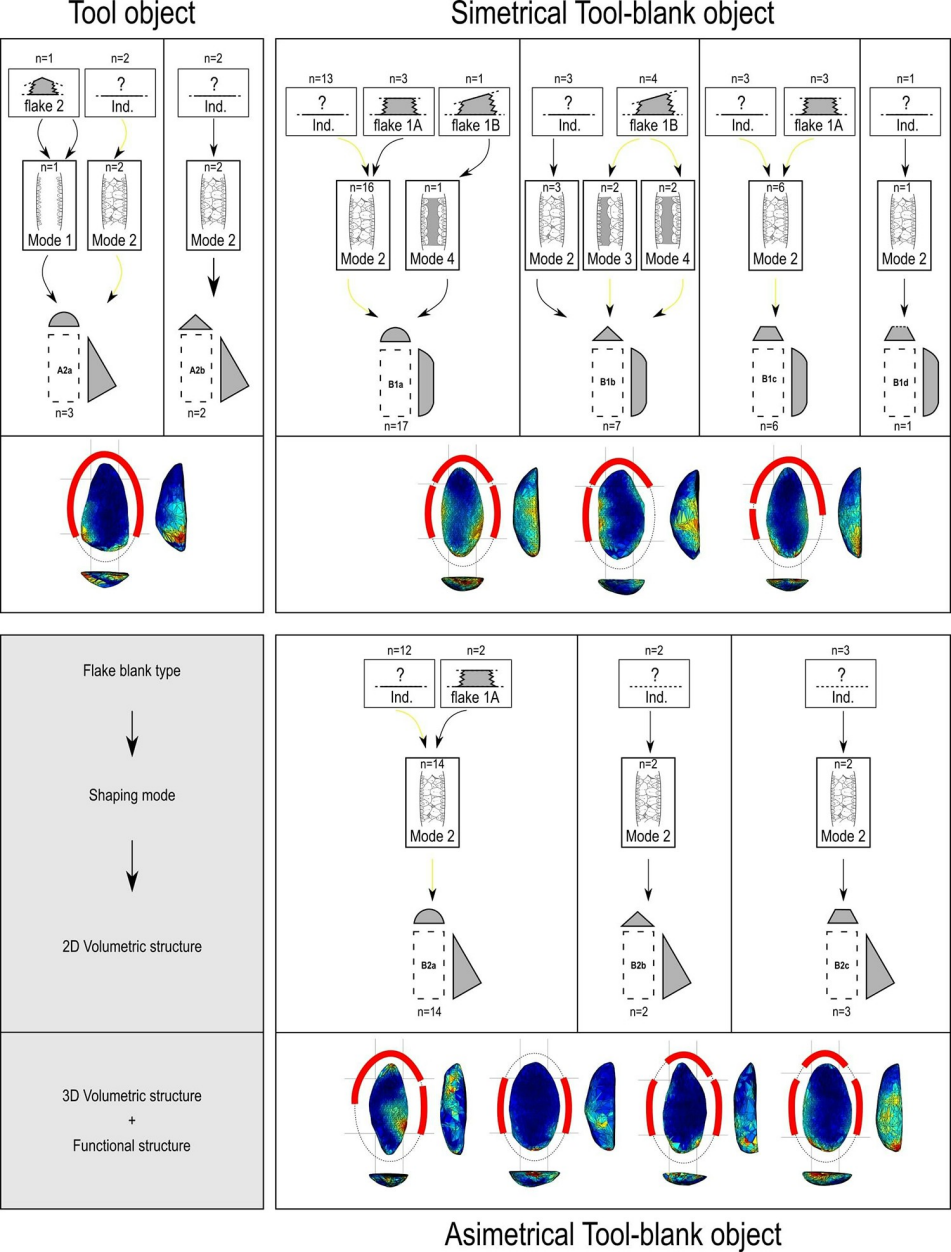
Synthesis of the techno-structural and potentially functional schemes determined at GO-Ni sites. In black, the most representative schemes at a qualitative level. In red lines, the Structural Units (SU) number and location. This figure was inspired by Lourdeau [41; Fig 70].

Fig 25 offers a comprehensive synthesis of our results in both 2D and 3D formats. The primary focus here is the characterization of unifacially shaped tool artifacts from GO-NI-01, utilizing a 3D techno-structural typology. Despite the presence of a relatively homogeneous concept underlying the manufacture of these tools in Central Brazil, their undeniable variability in silhouettes, structure, and functional potential has led to the identification of several sub-types. These sub-types, at times, have been interpreted similarly, yet they span a broad chronological period and cover a vast geographical area.

Our findings affirm the extensive variability within the unifacially shaped artifacts attributed to the Itapatica technocomplex of Central Brazil. Our research methodology aimed to move beyond their traditional typological classification, which primarily focuses on the outline, by shedding light on their structural history. Furthermore, we endeavored to minimize subjectivity in determining the geometry of sections, silhouettes, and volumetrics, surpassing the limitations of manual techno-structural analysis. By cross-referencing the identified techno-structural nine groups with data from 3D geometric morphometrics and the number and localization of Structural Units within their volumetric structure, we have been able to identify three major morpho-techno-structural families in GO-NI-01. This classification has potential implications for the technical functions of each edge and sub-volume of each piece:

- **Asymmetrical Profile Tool Object** (TSG: A2a and A2b), a group that represents 7.5% of the analyzed sample. These tools are primarily made on indeterminate flakes, produced through a shaping mode 2, which results in objects with a structure comprising at least three sub-volumes and a convergent SU extending to the base of the object. Our hypothesis is that this singular SU is likely transformative in nature. The variability of the 3D shapes in this group is mainly observed at the base of the object, coinciding with its thickest part.
- **Symmetrical Profile Tool-Blank Object** (TSG: B1a, B1b, B1c, and B1d), a group accounting for 52.2%. These tools are also primarily made on indeterminate flakes but with a significant presence of 1A and 1B flakes. Three shaping modes (2, 3, and 4) are associated with the formation of these flakes, though a clear preference for mode 2 is evident. These pieces exhibit a volumetric structure similar but not identical to the “limaces” of morphological typology. While generally consisting of a single volume, these pieces may also display up to three sub-volumes. The variability of the 3D shapes in this group is primarily in their mesial lateral edges, which coincide with their thickest part. Here, our hypothesis is that the SUs are ambivalent in nature, both transformative and prehensile. It is important to note that all these pieces always feature at least one convergent SU in the distal position, which may be accompanied by one or two lateral SUs, generally with a convex delineation in frontal view.
- **Asymmetrical Profile Tool-Blank Object** (TSG: B2a, B2b, and B2c), a group representing 40.3%. These tools are also primarily made on indeterminate flakes and subsequently shaped exclusively by mode 2. These pieces have a volumetric structure that can be divided into up to 4 sub-volumes. The variability of the 3D shapes in this group is seen in the distal, mesial, and basal parts, with the lateral edges being the least variable. It is crucial to note that all these pieces always have at least one lateral SU with a convex delineation, which may or may not be accompanied by a convex or convergent distal SU.

A noticeable dimensional gap exists between these groups, with the Tool-blank being more elongated than the Tool-object. On the other hand, all these groups could be further subdivided according to the morpho-techno-functional criteria of the identified SUs, but this is beyond the scope of this work. Thus, it is plausible that the diversification of unifacially shaped tools is underpinned by different tool concepts that are partially adapted to their volumetric structures and functional organization.

At this point, it is necessary to emphasize the primary methodological aspect of this work, namely the *a posteriori* functional characterization of the technical functions of each structural unit:

- Asymmetrical Profile Tool objects: a single transformative unit opposed to a prehensive unit.
- Symmetrical Profile Tool-Blank objects: a composite of a transformative unit recurrently in the apical position and one or two lateral transformative/prehensive units in an opposite oblique position to a prehensive unit that does not exhibit strong morpho-structural normalization.
- Asymmetrical Profile Tool-Blank objects: mostly associated with two lateral transformative/prehensive units adjacent to a basal prehensive unit without significant normalization.

Recent advancements in archaeological theory have highlighted the limitations of traditional cultural taxonomies, often criticized for their static and hierarchical nature. Reynold and Riede [139] provide a compelling critique of these frameworks, emphasizing the need for robust, dynamic, and context-sensitive approaches to grouping material culture. In alignment with these critiques, our study moves beyond traditional typological paradigms by adopting a 3D techno-structural approach to classify unifacially shaped tools. This method emphasizes structural history and volumetric organization over simplistic reliance on outlines or pre-defined types, addressing some of the fundamental issues Reynold and Riede [139] raise about the epistemological aims of cultural taxonomy. Specifically, we argue that integrating morphometric clusters and volumetric analyses provides a more nuanced understanding of tool variability, functionality, and adaptation. The findings at GO-NI-01 reveal extensive morphological and functional variability within tools attributed to the Itapatica technocomplex, supporting Reynold and Riede’s assertion that robust taxonomies must account for inter- and intra-assemblage variation. By combining 3D geometric morphometric data with detailed techno-structural analysis, we have identified three morpho-techno-structural families that reflect not only the artifacts’ manufacturing processes but also their adaptive roles within broader socio-ecological systems. This approach aligns with Reynold and Riede’s call for taxonomies that are open, revisable, and grounded in material analysis, enabling more meaningful comparisons across assemblages and regions. This is particularly important and urgent in the cultural taxonomy derived from stone tools in tropical South America.

### Functional implications of 3D techno-structural data

The 3D geometric morphometric analysis reveals significant morphological variability in certain Techno-Structural Groups (TSGs), such as B2c, B2a, and B1c. This variability likely reflects a complex interplay of factors, including diverse functional designs, varying degrees of experimentation, and broader cultural and social dynamics. For instance, differences in modes of cultural transmission, network density versus isolation, stylistic constraints, population density, and degrees of innovation could all contribute to the observed morphometric variability.

The differentiation between flake types and workshops may indicate unique technological processes or activities specific to these sites. The surface relief of the flakes, varying from strictly flat to convex, influences the potential use of the tools. The SDI offers insights into the intensity of shaping reduction of tool blanks. Morphostructural characteristics point to a standardized tool design approach, despite minor variations in blank shaping intensity. The presence and location of inflection and torsion lines suggest a potential functional predetermination in production techniques and structural design. Variations in their placement can significantly impact tool morphology and the location of the Structural Unit (SU). Different shaping modes, coupled with relatively homogeneous SDI, hint at targeted functional intentions for the tools. The distribution of mass and shape, influenced by the intensity of shaping mode, is crucial for determining a tool’s suitability for specific tasks. However, the distribution of mass and shape, shaped by the intensity of the reduction process, may also reflect broader socio-cultural influences, making it a critical factor for understanding both the technological and cultural dimensions of tool production.

This study corroborates the strong relationship between shaping methods and intended tool structure, as previously indicated by Lourdeau [41, 59]. In the sample studied, resharpening does not significantly contribute to the variability of 3D shapes. This is likely because the studied pieces were not in a state of structural denaturation *sensu* Lourdeau [41], hence the morpho-structural deviation from the original design is minimal. Further studies with more reconfigured and denatured pieces are necessary to deepen understanding of the role of this attribute. The location of the SU is crucial for understanding tool design and functionality. Variations in SU location point to different ergonomic considerations and functional adaptations. This aspect seems to have significantly influenced the techno-structural variability of the GO-NI-01 site assemblage, as demonstrated by various 3D geometries.

The application of Bordes’ typology, while providing a traditional and widely known framework for understanding tool variety, in our study suggests more of a limitation in grasping tool design than a true continuum in design evolution and functional roles. Although this typology is specifically valuable for the Mousterian of southwest France, we have invoked it in this work due to its regular use as a "universal" typological reference in prehistoric lithic technology. At no point do we intend to extrapolate it for understanding the sample under study. Based on the foregoing, our hypothesis is partially confirmed. Tool function indeed better explains the variability of 3D shapes in GO-NI-01 open-air sites, at least in the workshops analyzed. However, despite a statistically significant difference between shapes made of different raw materials, we remain cautious due to the lack of more analyses with greater representation of silicified sandstone or other raw materials.

### Implications of the structural and functional variability of Central Brazil unifacial tools

If the internal techno-structural and potentially functional variability of tools at GO-NI-01 can be attributed to the reasons discussed above, what implications does this have for a broader regional understanding of the so-called “unifacially shaped tools”? The limitations of a strict typological definition are evident, as limaces often overlap with other types in both 3D shapes and techno-structural features. The identified Techno-Structural Groups (TSG) are represented differently within the tool types, but theoretically, every TSG can be achieved using various types of blanks and shaping/retouching modalities. This is also evident in our case study, where no exclusive concept for Limace exists, though certain clear preferences are noted. In this regard, the only commonality shared by the tool groups identified in this study is the unifacial shaping. In GO-NI-01, not all PFUFP (Unifacially shaped artefact with a flat face) are elongated and thick, as evidenced by the group of asymmetrical tool objects. At this site, not all PFUFPs are designed to support at least one transformative SU at the apical end. Furthermore, resharpening and reconfiguration do not seem to have a significant presence at this site, though this might be due to a specific facet of the site, possibly as a quarry-workshop. Thus, in the analyzed sample, GO-NI-01 appears to encompass the PFUFP phenomenon but significantly exceeds it. This fact, along with the existence of five different reduction sequences, allows us to propose a particular specificity of the unifacial phenomenon at GO-NI-01. What does this mean in terms of its affiliation with the Itaparica technocomplex? Some of the tools in our sample can be easily associated with the techno-functional groups of unifacially shaped artifacts in GO-JA-01, Boqueirão da Pedra Furada (Serra Talhada I layer), and Pica-Pau, as determined by Lourdeau [59], but there are others that are not present, such as the symmetrical tool objects that are absent in GO-NI-01. However, comparison is challenging due to the limitations of 2D descriptions. Moreover, the same methodological procedure that we have used here needs to be applied to the flake, pebble, and slab tools of GO-NI-01 to observe how they associate with the unifacially shaped artifacts. Other detailed techno-structural studies from Central Brazil [43, 47] also present some of the tools identified in this work. This suggests the need for a microregional technography at these sites to progress towards a comparative technology, as previous studies have rightly pointed out and advanced in this direction.

## Conclusion

This research on Holocene unifacial tools from the GO-NI-01 sites in Central Brazil, particularly within the Itaparica technocomplex phenomenon, has provided insights into the technological strategies and adaptations of early human populations to their functional needs. Our approach, combining 3D geometric morphometry with techno-structural analysis, has illuminated the delicate interplay between raw material availability, functional necessities, and ergonomic factors in tool crafting. The discovery of a broad spectrum of 3D tool geometries within the identified techno-structural groups (TSGs) indicates a diverse, adaptive, and flexible tool-shaping strategy. This diversity challenges traditional morpho-typological classifications and highlights a possible new range of functional objectives stemming from an open-air site context. This tool variability is also accompanied by an uncommon technological context in Itaparica shelter sites, likely reflecting a unique quarry-workshop setting. Moreover, the observed variability in tool 3D shapes suggests a sophisticated, probably function-driven approach to tool production, likely tailored to specific functional requirements and ergonomic considerations, rather than constrained by raw material availability, production methods, or strict morphological templates. Our analysis of the unifacially shape tools type reveals a certain fluidity in its definition, encompassing multiple sub-types that suggest different volumetric and potential functional organizations. These distinctions are based on variations in morphology, structural units, dimensions, and other factors. In summary, this study enhances our understanding of lithic technology among early South American populations and contributes to the broader narrative of human adaptation and innovation during the Holocene. If this type of tool morpho-structure appears at some point in South American prehistory, it is because it was necessary for human groups in a particular environment with specific requirements. The case of GO-NI-01 suggests that perhaps we should consider more of an Itaparica spectrum rather than an Itaparica phenomenon.

By unraveling the intricate relationship between functional design and morphology, this research adds a new dimension to our comprehension of prehistoric tool-making and usage in Central Brazil. Future research, especially with a broader comparative sample and the integration of experimental and use-wear approaches, promises to further unravel the complexities of early human technological strategies in this region and beyond.

## Supporting information

S1 Table Brazilian sites with and without unifacial shaping. (XLSX)

S2 Table Manual 3D analysis database. (XLSX)

S3 Table Confidence intervals for Mann-Whitney U test comparisons of principal measurements of unifacially shaped artifacts. (CSV)
